# Development and exploration of novel substituted thiosemicarbazones as inhibitors of aldose reductase via in vitro analysis and computational study

**DOI:** 10.1038/s41598-022-09658-z

**Published:** 2022-04-06

**Authors:** Aqeel Imran, Muhammad Tariq Shehzad, Syed Jawad Ali Shah, Taha al Adhami, Mark Laws, Khondaker Miraz Rahman, Rima D. Alharthy, Imtiaz Ali Khan, Zahid Shafiq, Jamshed Iqbal

**Affiliations:** 1 Center for Advanced Drug Research, COMSATS University Islamabad, Abbottabad Campus, Abbottabad, 22060 Pakistan; 2 Department of Pharmacy, COMSATS University Islamabad, Abbottabad Campus, Abbottabad, 22060 Pakistan; 3 Institute of Chemical Sciences, Bahauddin Zakariya University, Multan, 60800 Pakistan; 4 School of Cancer and Pharmaceutical Sciences, King’s College London, Franklin-Wilkins Building, 150 Stamford Street, London, SE1 9NH United Kingdom; 5 Chemistry Department, Faculty of Science and Arts, King Abdulaziz University, Rabigh, 21911 Saudi Arabia; 6 Department of Entomology, Agricultural University, Peshawar, 25130 Khyber Pakhtunkhwa Pakistan

**Keywords:** Biochemistry, Drug discovery

## Abstract

The role of aldose reductase (ALR2) in causing diabetic complications is well-studied, with overactivity of ALR2 in the hyperglycemic state leading to an accumulation of intracellular sorbitol, depletion of cytoplasmic NADPH and oxidative stress and causing a variety of different conditions including retinopathy, nephropathy, neuropathy and cardiovascular disorders. While previous efforts have sought to develop inhibitors of this enzyme in order to combat diabetic complications, non-selective inhibition of both ALR2 and the homologous enzyme aldehyde reductase (ALR1) has led to poor toxicity profiles, with no drugs targeting ALR2 currently approved for therapeutic use in the Western world. In the current study, we have synthesized a series of N-substituted thiosemicarbazones with added phenolic moieties, of which compound **3m** displayed strong and selective ALR2 inhibitory activity *in vitro* (IC_50_ 1.18 µM) as well as promising antioxidant activity (75.95% free radical scavenging activity). The target binding modes of **3m** were studied *via* molecular docking studies and stable interactions with ALR2 were inferred through molecular dynamics simulations. We thus report the N-substituted thiosemicarbazones as promising drug candidates for selective inhibition of ALR2 and possible treatment of diabetic complications.

## Introduction

**Figure 1:**
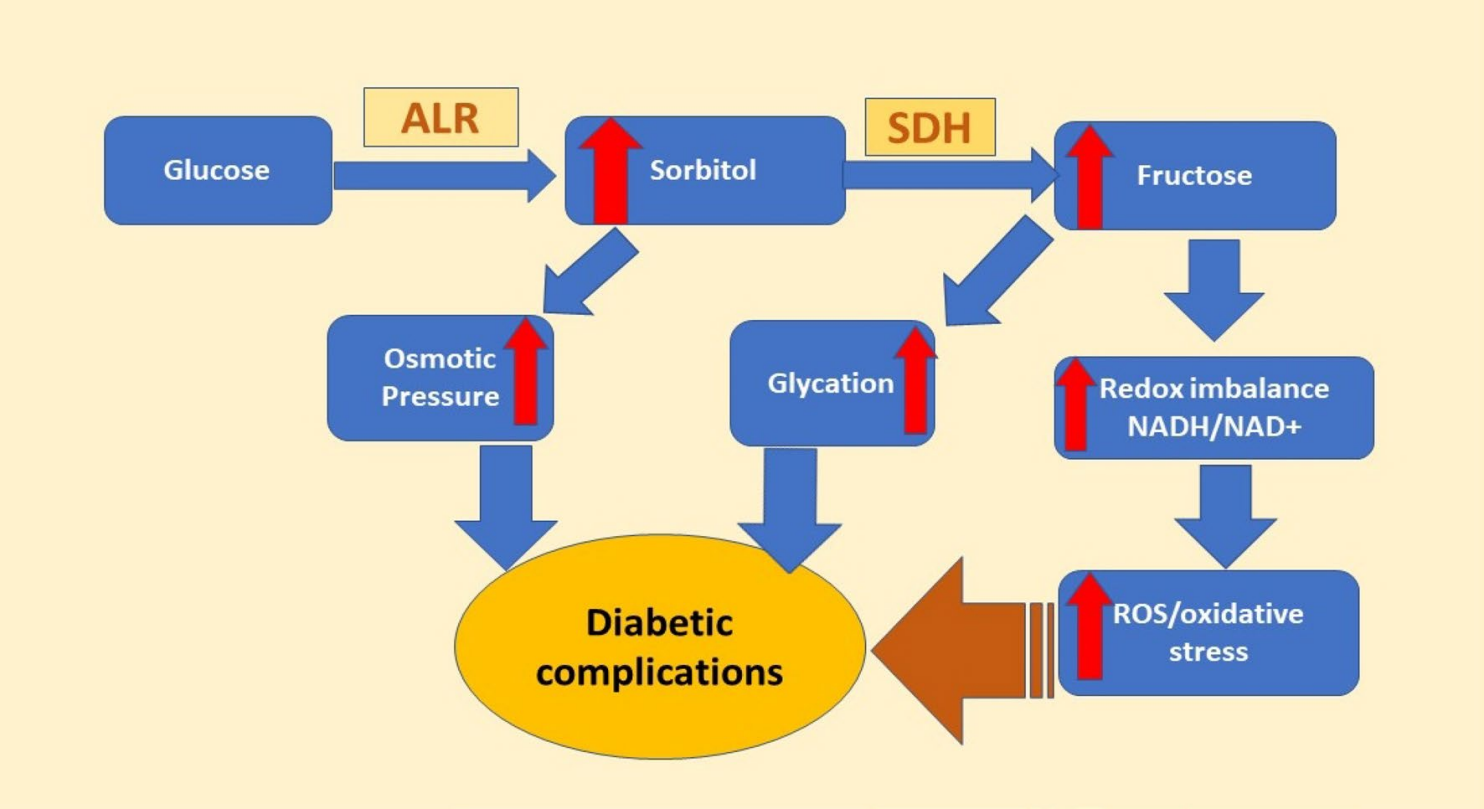
The polyol pathway. Abbreviations: *ALR* aldose reductase, *SDH* sorbitol dehydrogenase.

The enzyme aldose reductase (ALR2, AKR1B1; EC 1.1.1.21) is a member of the aldo-keto reductase superfamily^1^ that catalyzes the reduction of a wide variety of toxic aldehydic substrates to the corresponding, less toxic alcohols. It also catalyzes the reduction of glucose to sorbitol in the presence of NADPH as the first (and rate-determining) step in the polyol pathway (Fig. 1). This pathway converts glucose to fructose in a two-step process; another enzyme, sorbitol dehydrogenase (SDH), mediates the second step in which sorbitol is converted to fructose and NAD^+^ reduced to NADH^2^. In normoglycemic conditions, glucose exists largely in the form of glucose-6-phosphate and undergoes glycolysis, with the poor affinity of the phosphorylated species for ALR2 meaning that only the small fraction of unphosphorylated glucose present can enter the polyol pathway^3^. During a state of hyperglycemia, however, elevated levels of blood glucose drive increased flux through the polyol pathway, resulting in the intracellular accumulation of sorbitol and the depletion of cytoplasmic NADPH and causing oxidative stress and reduced glutathione levels. This culminates in the emergence and progression of diabetic complications including cataracts, neuropathy, retinopathy, nephropathy, atherosclerosis and others cardiovascular disorders^4,5^. The connection between ALR2 and diabetic complications has driven efforts to develop ALR2 inhibitors as treatments and several such drugs have been developed synthetically and identified from natural sources^6–8^.

The challenging aspect of the design and development of ALR2 inhibitors is ensuring selectivity of inhibition since another enzyme belonging to the aldo-keto reductase superfamily, aldehyde reductase (ALR1, AKR1A1, EC 1.1.1.2), shares 65% homology with ALR2. This isozyme ALR1 is involved in the reduction of toxic aldehydes such as methylglyoxal and 3-oxyglucosazone to their less toxic alcoholic counterparts and such kind of reduction was dependent upon cofactor NADPH^9^. Non-selective inhibition of both isozymes is thought to be a major contributor to the toxicity issues that have plagued previous efforts to develop ALR2 inhibitors^10^, with the only currently available approved treatment for diabetic complications being epalrestat in Japan, China and India^11,12^. Therefore, the development of a new generation of more selective ALR2 inhibitors with fewer side effects, enhanced tissue permeability and reduced toxicity must be prioritized.

**Figure 2:**
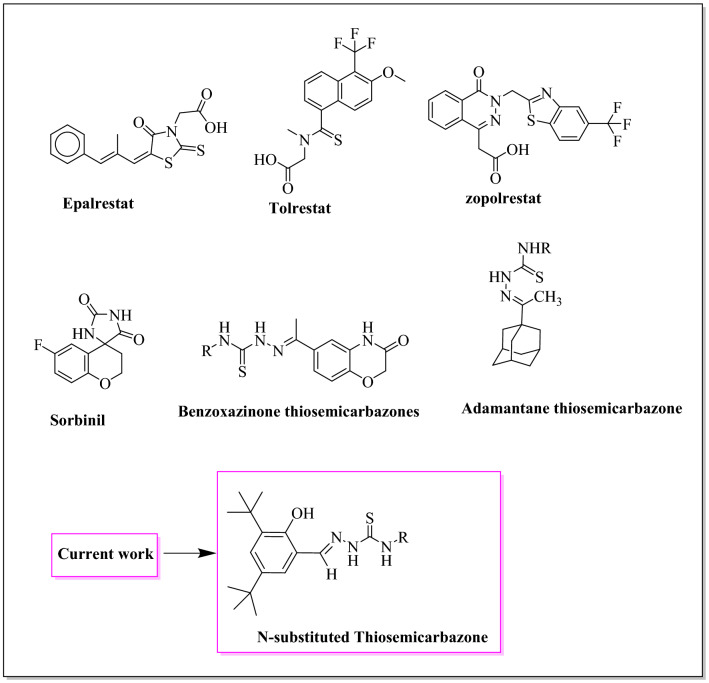
Some reported aldose reductase inhibitors and the N-substituted thiosemicarbazones reported herein.

In our recent research studies, adamantyl-based and benzoxazinone-based thiosemicarbazones have shown potent and selective inhibition of ALR2^7,13^. Considering the ALR2 inhibitory potential of the thiosemicarbazone scaffold, the present study involved the design and development of various N-substituted thiosemicarbazones for testing against ALR2 (Fig. 2). Additionally, the incorporation of phenolic moieties was hoped to generate compounds with antioxidant activities^14^ and thus dual-action drug candidates for the treatment of diabetic complications.

## Results and discussion

### Chemistry

**Scheme 1:**
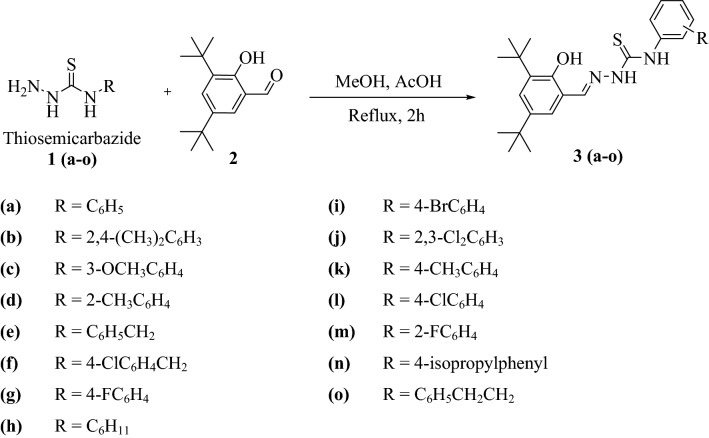
Synthesis of substituted thiosemicarbazone derivatives **3a-o**.

The synthesis of thiosemicarbazone hybrids **3a-o** was carried out as detailed in Scheme 1. The starting thiosemicarbazide compounds **1a-o** were prepared by reaction of the corresponding isothiocyanate with hydrazine in an ethanol/water solution as reported previously by da Silva *et al*. ^15^. The thiosemicarbazone derivatives **3a-o** were synthesized through condensation of the appropriate N^4^-substituted thiosemicarbazide (**1a-o**) with 3,5-di-tert-butyl-2-hydroxybenzaldehyde (**2**) in methanol with a catalytic quantity of glacial acetic acid. Reaction conditions were optimized by treating phenyl thiosemicarbazide (**1a**) (1 mmol) with 3,5-di-tert-butyl-2-hydroxybenzaldehyde (**2**) (1 mmol) using different solvents such as ethanol, methanol, DCM, THF and DMSO. Methanol was established as the best solvent for the reaction while glacial acetic acid (1-2 drops) was identified as an effective catalyst. A variety of target N^4^-substituted thiosemicarbazones were obtained in pure form in good to excellent yields (79–90%) *via* recrystallization from ethanol.

The structures of compounds **3a-o** were confirmed by CHN analysis and various spectroscopic techniques. The infrared (IR) spectra showed absorption bands in the range of 1547–1580 cm^−1^ due to the new azomethine linkage (C=N) while C=S stretching was observed in the 1189–1245 cm^−1^ range. NH bands appeared in the 3218–3328 cm^−1^ region while absorption in the 3398–3450 cm^−1^ range indicated the presence of an OH functional group. In ^1^H NMR, the methyl protons of two tertiary butyl groups resonated at 1.27–1.34 ppm and 1.40–1.48 ppm, respectively, while the proton of the azomethine moiety (N=CH) appeared as a singlet in the 8.06–8.39 ppm range. Similarly, the NH-CS proton appeared as a singlet in the range of 7.42–10.15 ppm, whereas the NH-N proton was also observed as a singlet in the 9.75–11.18 ppm region. In thiosemicarbazone **3h**, a derivative bearing a cyclohexyl group, a doublet was observed for NH-CS at 7.42 ppm. The most downfield signal observed was consistently attributed to the OH functional group, appearing in the region of 9.87–11.91 ppm. The structures of compounds **3a-o** were also confirmed by ^13^C NMR spectroscopy. The methyl carbons of the tertiary butyl moiety were found to resonate in the region of 29.40–31.74 ppm while methyl carbons directly attached to the second aromatic ring appeared at 17.82–20.71 ppm. The carbon of the CH=N group appeared in the range of 147.25–145.65 ppm while the most downfield signal (176–179.57 ppm) was attributed to the C=S group carbon. In LC-MS spectra, molecular ion peaks of the compounds appeared as [M+H]^+^ which were consistent with the molecular weights of the synthesized derivatives.

### Enzyme inhibition and structure–activity relationship (SAR)

Compounds **3a-o** were tested against ALR2 to evaluate their inhibitory potential against aldose reductase whereas selectivity was determined via performing ALR1 inhibition assay. For the enzyme inhibition assay, ALR2 and ALR1 were extracted from bovine sources (ALR2 from eyes and ALR1 from kidneys). Moreover, inhibitions studies were also carried out on the expressed ALR2 (human AKR1B1) and inhibition data for ALR2- bovine source and expressed enzyme was compared and analyzed. The protocols for enzyme extraction and expression in the bacterial system are included in the section of “Supporting information”. The compounds which demonstrated greater than 50% target inhibition at 100 µM were further investigated to determine their IC_50_ values, summarized in Table 1. For the purpose of comparison and validation of enzyme inhibition data, these compounds were tested against human AKR1B1 (hAKR1B1) expressed in *E. coli* BL21 (DE3). We found a similar pattern of enzyme inhibition for the synthesized compounds against extracted enzyme (ALR2) as well as expressed enzyme (hAKR1B1). Enzyme inhibition data (against ALR2, hAKR1B1 and ALR1) is summarized in Table 1.

**Table 1:** IC_50_ values for ALR1 and ALR2 inhibition.


Compounds code	IC_50_ ± SEM (ALR2) µM^a^/ I (%, 100 µM)^b^	IC_50_ ± SEM (AKR1B1) µM^a^/I(%, 100 µM)^b^	IC_50_ ± SEM (ALR1) µM^a^/ I (%, 100 µM)^b^
**3a**	2.99 ± 0.0021	3.89 ± 0.0034	2.83 ± 0.0034
**3b**	30.72%	27.43%	31.62%
**3c**	4.49 ± 0.0034	3.27 ± 0.0035	4.21 ± 0.0021
**3d**	1.15%	3.56%	3.13%
**3e**	9.56%	12.34%	11.39%
**3f**	3.12 ± 0.0031	3.04 ± 0.0034	4.27%
**3g**	2.38 ± 0.0028	3.95 ± 0.0038	10.54%
**3h**	39.13%	37.98%	4.27%
**3i**	25.50%	22.26%	10.54%
**3j**	4.01 ± 0.0021	2.75 ± 0.003	5.12%
**3k**	44.63%	41.22%	25.07%
**3l**	**1.46 ± 0.005** **81.54%**^**b**^	1.65 ± 0.004	26.21%
**3m**	**1.18 ± 0.0034** **85.08%**^**b**^	1.36 ± 0.004	5.98%
**3n**	24.63%	22.56%	14.81%
**3o**	21.73%	24.86%	29.91%
**Sorbinil**^**c**^	2.18 ± 0.002	1.44 ± 0.023	–
**Valproic acid**^**d**^	–	–	49.31 ± 0.005

^a^The IC_50_ value (the half-maximal inhibitory concentration) is the concentration of drug required to decrease enzyme activity by 50%.

^b^The percentage inhibition for enzymes measured at 100 µM of inhibitor concentration.

^c^Sorbinil is a standard inhibitor of ALR2.

^d^Valproic acid is a standard inhibitor of ALR1. Results are mean values ±SEM based on three measurements.

Synthesized compounds’ codes were written in bold text whereas the most significant and selective inhibitor’s values were made bold upon suggestion of reviewer’s comments.

The *in vitro* reduction of substrate DL-glyceraldehyde by ALR2 in the presence of the synthesized compounds was assessed and compared to control inhibitor sorbinil. While **3a**, **3c**, **3f**, **3g**, **3j**, **3l** and **3m** all showed strong inhibition of ALR2 (IC_50_ range 1.18–4.49 µM), only **3f**, **3g**, **3j**, **3l** and **3m** were selective for ALR2 (4.27–26.21% inhibition of ALR1), with **3a** and **3c** demonstrating non-selective inhibition of both ALR1 and ALR2 (**3a** ALR2 IC_50_ 2.99 µM, ALR1 IC_50_ 2.83 µM). Notable selective ALR2 inhibitors identified were **3l** and **3m**, with each showing strong and selective inhibition of ALR2 (IC_50_ values of 1.46 µM and 1.18 µM, respectively) and low to moderate inhibition of ALR1 (26.21% and 5.98% inhibition, respectively). In contrast, **3b**, **3d**, **3e**, **3h**, **3i**, **3k**, **3n** and **3o** all showed low to moderate inhibition of both ALR1 and ALR2.

From these data, a structure–activity relationship (SAR) for the N-substituted thiosemicarbazones with respect to both ALR1 and ALR2 inhibition was established. For all synthesized compounds, the NH group of the thiosemicarbazone was functionalized with either a phenyl or benzyl group. These aromatic rings were substituted with different electron withdrawing or electron donating groups at various positions. The phenyl ring of **3a** was associated with non-selective inhibition of both ALR1 and ALR2, with the addition of methyl substituents at the *ortho* and/or *para* positions (**3b**, **3d**, **3k**) resulting in a decrease in inhibition of both enzymes (≤44.63% for ALR2, ≤31.62% for ALR1). A *meta* methoxy group (**3c**) resulted in strong inhibition of both enzymes (ALR2 IC_50_ 4.49 µM, ALR1 IC_50_ 4.21 µM) while incorporation of electron withdrawing fluorine (**3g**) and chlorine (**3l**) atoms at the *para* position of the phenyl ring, as well as the benzyl group (**3f**), enhanced inhibition of ALR2 and caused selective inhibition (**3f** ALR2 IC_50_ 3.12 µM, 4.27% inhibition of ALR1). Other compounds possessing fluorine and chlorine substitutions at *ortho* and *meta* positions on the phenyl ring (**3j**, **3m**) also showed selective inhibition of ALR2 (**3m** ALR2 IC_50_ 1.18 µM, 5.98% inhibition of ALR1). Bromine (**3i**) and isopropyl (**3n**) substitutions at the phenyl ring *para* position, as well as cyclohexyl (**3h**), benzyl (**3e**) and phenethyl (**3o**) analogs of **3a**, all resulted in significant loss of both ALR1 and ALR2 inhibition (≤39.13% for ALR2, ≤29.91% for ALR1).

### DPPH radical scavenging activity

**Figure 3:**
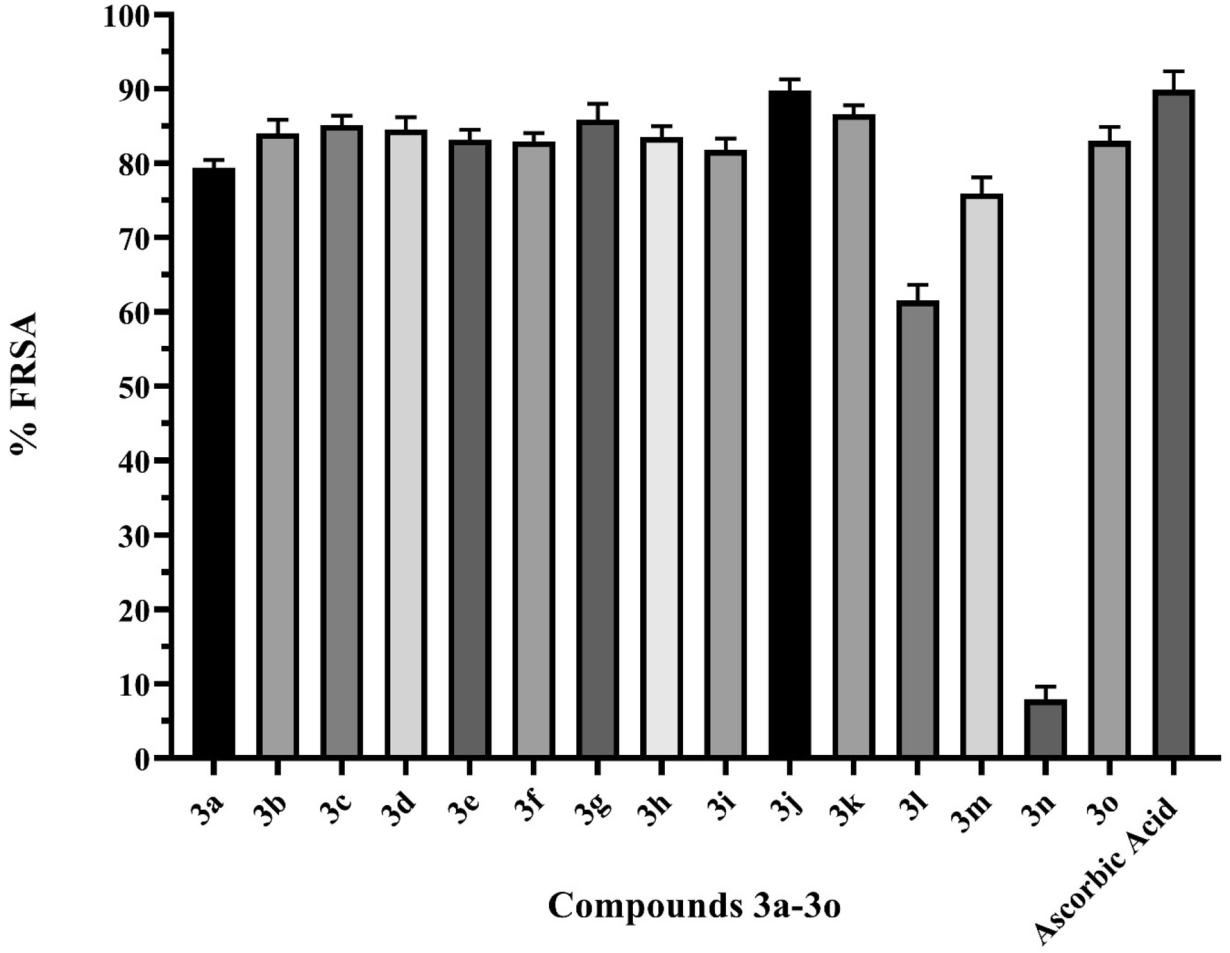
DPPH free radical scavenging activity (FRSA). Ascorbic acid was used as a positive control.

DPPH (2,2-diphenyl-1-picrylhydrazyl) was used to explore the free radical quenching ability of **3a**-**o** so as to provide a gauge of their antioxidant potential. The experiment was conducted according to a previously reported protocol with slight modifications^16^. Percent free radical scavenging activities (% FRSA) of **3a**-**o** were determined by spectrophotometric analysis at 517 nm using a homogenous mixture of methanolic DPPH (0.025 mg/mL) and a 100 µM solution of the tested compound, with ascorbic acid used as a positive control (Fig. 3). The majority of the compounds showed strong antioxidant potential (60–90% FRSA), though **3n** was notably weak (<10% FRSA). **3j** exhibited 89.56% FRSA, a value greater than that of the positive control (Table 2).

**Table 2:** The percent free radical scavenging activity of **3a**-**o**.

Compound	% FRSA	Compound	% FRSA
**3a**	79.41	**3i**	81.80
**3b**	84.05	**3j**	89.75
**3c**	85.16	**3k**	86.60
**3d**	84.58	**3l**	61.55
**3e**	83.20	**3m**	75.95
**3f**	82.98	**3n**	7.91
**3g**	85.89	**3o**	83.02
**3h**	83.54	**Ascorbic acid**	89.88

DPPH free radical scavenging activity expressed as percent free radical scavenging activity (% FRSA).

Synthesized compounds’ codes were written in bold text.

### Molecular docking results

*In vitro* testing identified **3l** and **3m** as promising selective inhibitors of ALR2, whereas **3a** and **3c** showed non-selective inhibition of both ALR2 and ALR1. Therefore, molecular docking studies were carried out for compound **3m** and their interactions with the amino acid residues of the AKR1B1 active site analyzed. To perform this docking analysis, the crystal structures of AKR1B1 (1US0)^17^ and AKR1A1 (3FX4)^18^ were downloaded from the Protein Data Bank and docking protocols from a recent study conducted within the group were used^13^.

**Figure 4:**
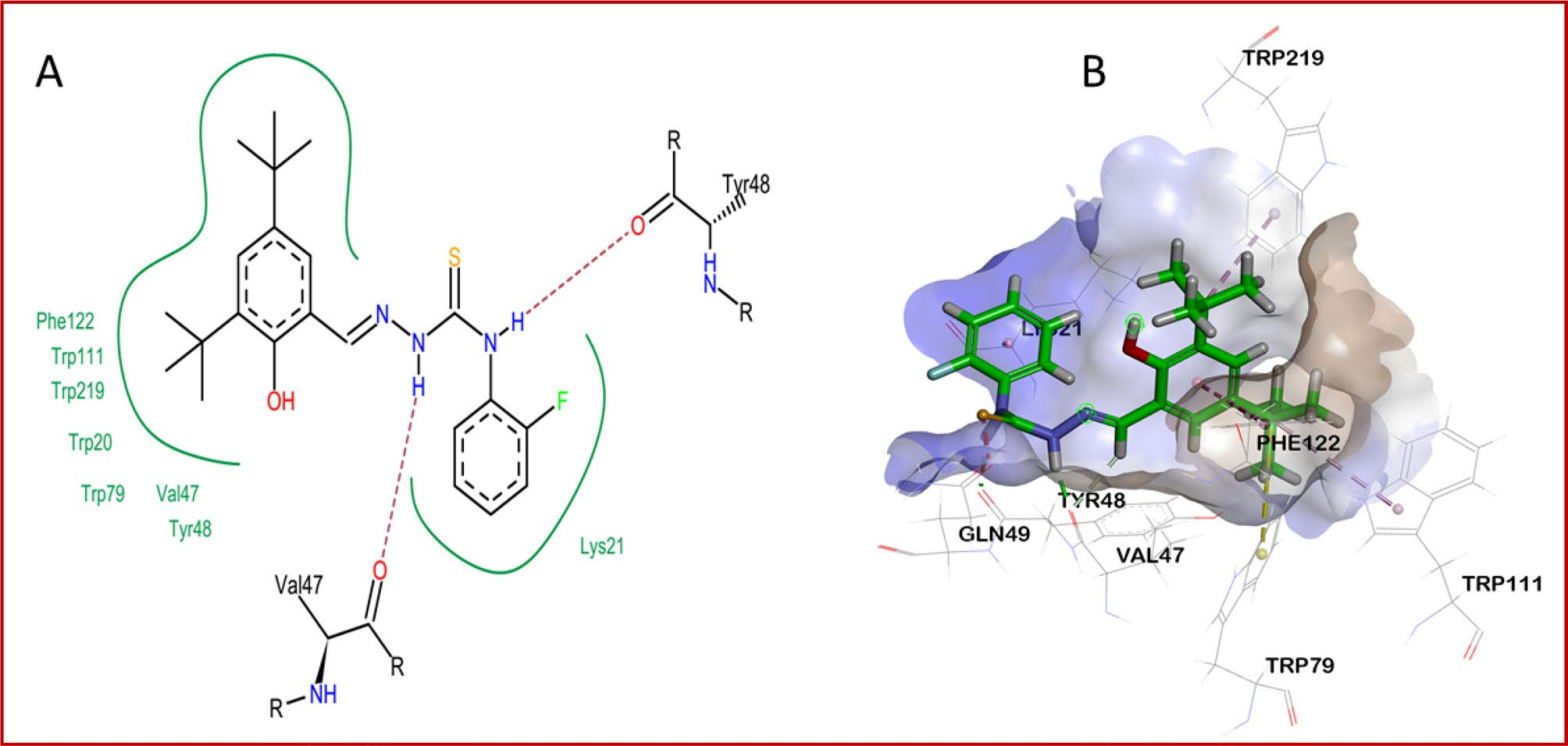
Two- and three-dimensional views of the interactions of **3m** within the active site of AKR1B1 are shown. (**A**) Two dimensional interactions show the involvement of Val47 and Tyr48 in hydrogen bond formation with the inhibitor. (**B**) Overall three-dimensional interaction poses showing all interacting amino acids residues within the active site.

Before docking of the synthesized inhibitor, redocking was done with inhibitor LDT320 (co-crystallized with ALR2) for the purpose of validation. To achieve reproducible docking results, the root-mean-square deviation (RMSD) value of co-crystallized inhibitor was found 0.69Å while using docking software (LeadIT). The most active inhibitor of ALR2 identified, **3m**, was selected for docking and it was observed that **3m** showed a similar binding orientation and conformation within the active pocket of ALR2 as the co-crystallized inhibitor^17^. **3m** was also docked against the active pocket of ALR1 and its interactions compared to those formed with ALR2. Two- and three-dimensional views of the interactions of **3m** within the active site of ALR2 are shown in Fig. 4; notable interactions included hydrogen bonds between Val47/Tyr48 and the hydrogen atoms of the two thiosemicarbazone -NH moieties, a π-alkyl interaction with Trp111 and van der Waals interactions with several hydrophobic residues including Trp20, Lys21, Phe121, Trp219 and Leu300.

### Molecular dynamics simulations

To revalidate the aforementioned docking results, molecular dynamic simulations of **3m** in the ALR2 active site in the presence of cofactor (NADPH) were carried out. The enzyme-cofactor-inhibitor system was solvated in a cubic PBC water box and the overall charge was neutralized using the counterions. The system was observed up to 50 ns.

**Figure 5:**
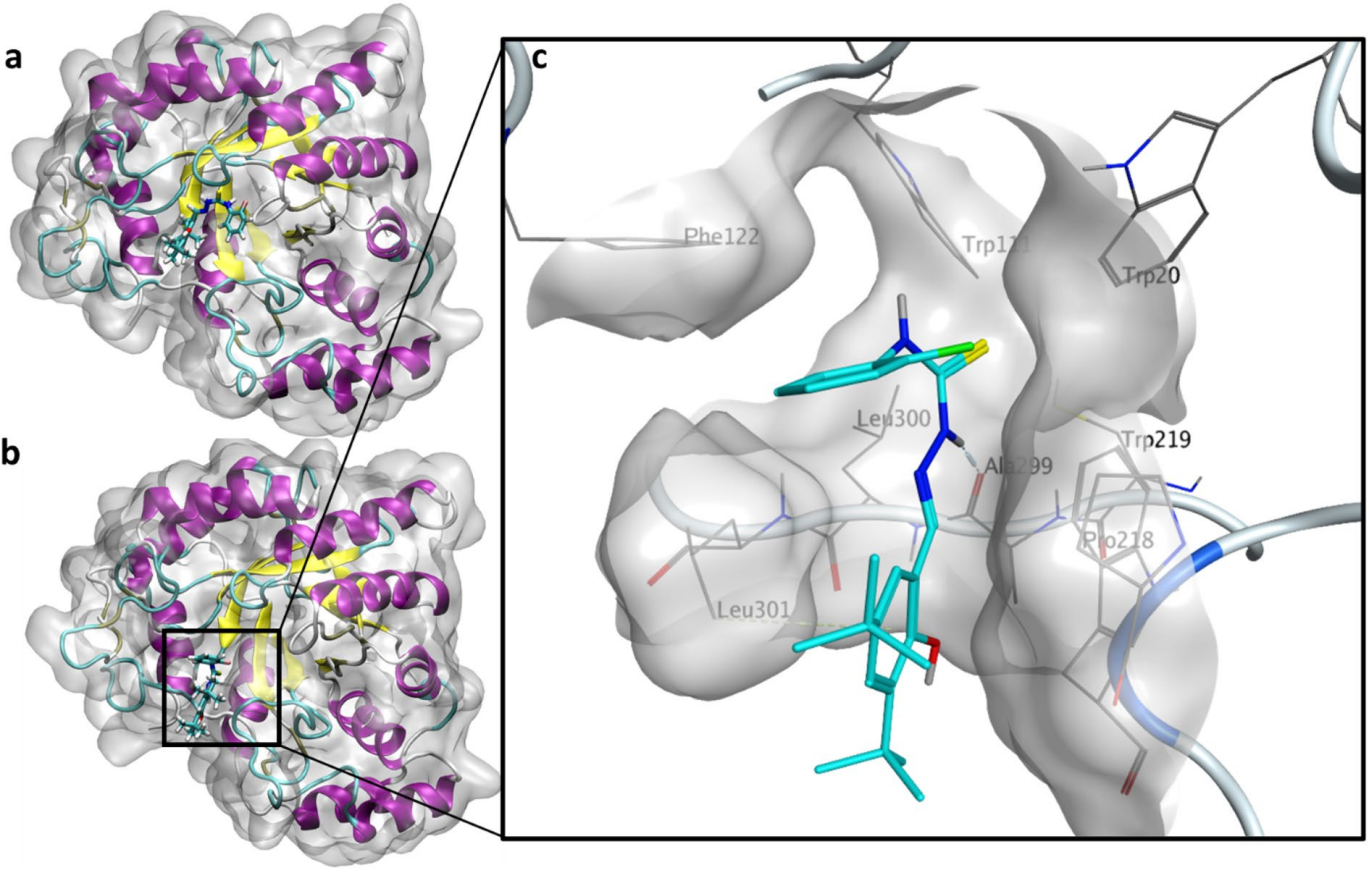
Simulated poses of **3m** inside ALR2. (**a**) Initial docked pose of **3m** inside ALR2. (**b**) Snapshot of **3m** at the 50 ns timepoint. (**c**) Interactions of **3m** with the ALR2 enzyme.

The RMSD values of the protein backbone, cofactor and inhibitor (**3m**) were observed to determine any drastic change; there was no considerable change in the RMSD of the protein backbone and cofactor. However, the RMSD of **3m** showed drastic fluctuations from the start of the simulation up until 20 ns, after which it remained constant. Upon visual inspection of the 50 ns trajectory, it was clear that the selected docked conformation was not very stable, drifting from its initial position within the anionic pocket (Fig. 5a) to the specificity pocket (Fig. 5b). The average structure from the trajectory after 20 ns of MD simulations was observed to occupy the specificity defining pocket. **3m** formed several interactions with residues within the pocket, including a hydrogen bond between the hydrogen atom of one of the -NH moieties and Ala299, an arene-H interaction with Leu301 and hydrophobic interactions with Trp20, Phe122, Pro218, Trp219 and Leu300 (Fig. 5c). These results provide a molecular-level rationale for the specificity of **3m** as observed in the *in vitro* enzyme inhibition assay.

**Figure 6:**
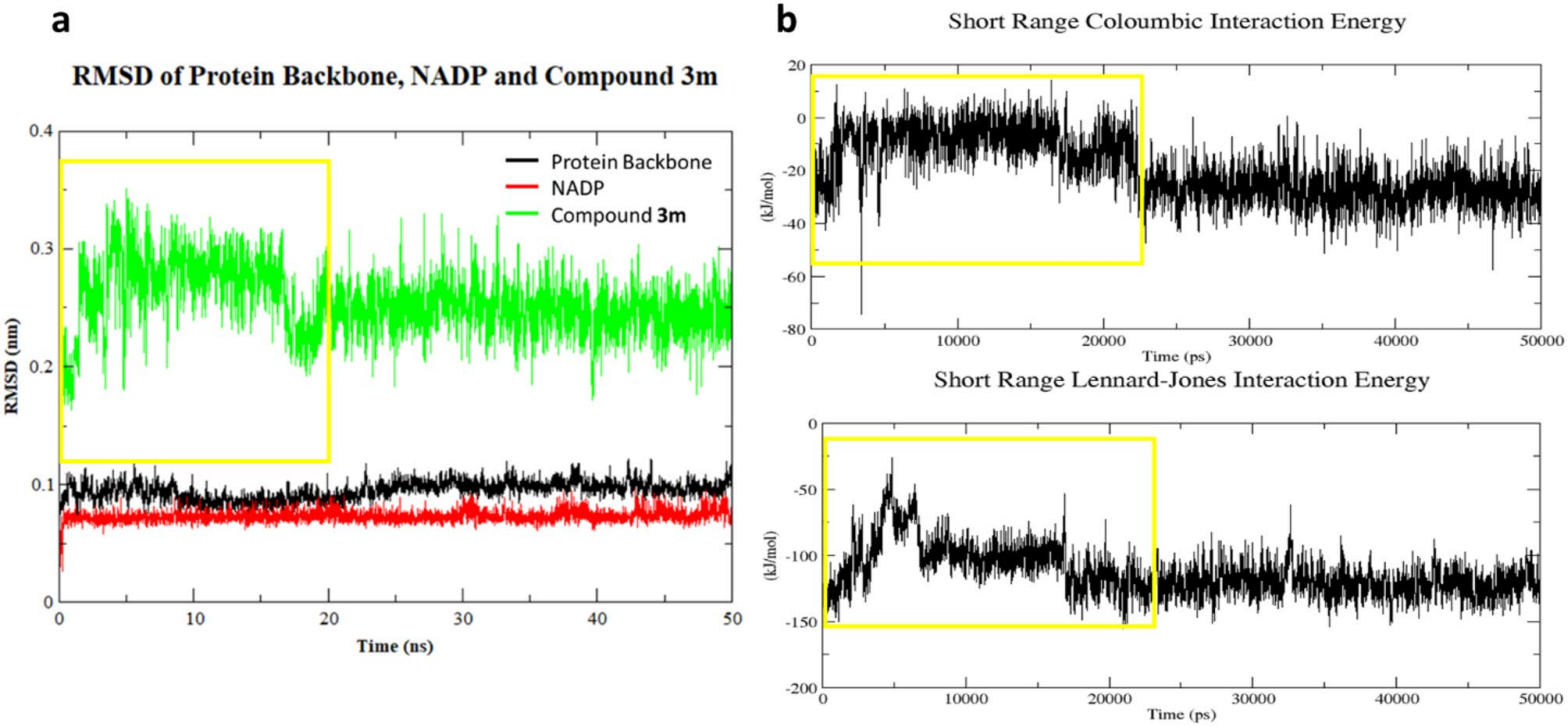
Root-mean-square deviation (RMSD) and short-range interaction energy profiles for **3m** in the ALR2 active site. (**a**) RMSD of protein backbone, cofactor and **3m**. (**b**) Short-range coulombic and Lennard-Jones interactions.

To further investigate the drastic fluctuations in RMSD values seen for **3m**, the short-range coulombic and Lennard-Jones (LJ) interactions were plotted. Fluctuations in RMSD values coincided with fluctuations in both the short-range coulombic and LJ interactions, occurring until around 20 ns. The higher values of short-range coulombic and LJ interaction energy mean **3m** is not as stable as after 20 ns and therefore the most probable pose is regarded as the one obtained after running the 20 ns simulations (see Fig. 6).

### Pharmacokinetic profile and ADME evaluation

**Figure 7:**
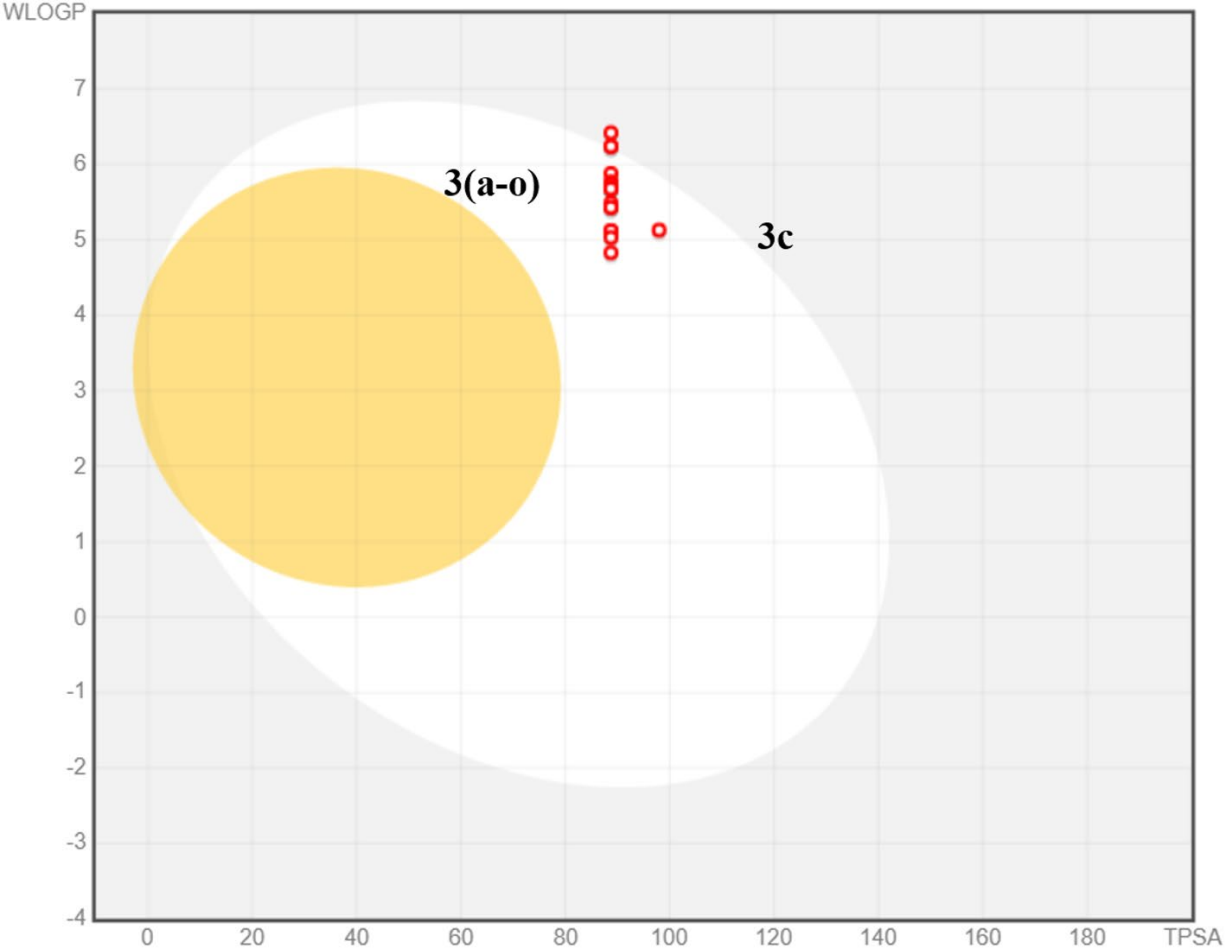
Boiled-egg plot for substituted-thiosemicarbazones for ADME evaluation.

Absorption, distribution, metabolism and excretion (ADME) studies for **3a**-**o** were carried out using an *in silico* method (SwissADME) that uses various algorithms to predict ADME parameters. The results of this analysis (Table 3) were used to draw a brain or intestinal estimated permeation (BOILED‐Egg) plot (Fig. 7), a plot of topological polar surface area (TPSA) against Wildman-Crippen partition coefficient (WLOGP) that predicts gastrointestinal absorption (white area) and blood-brain barrier permeation (yellow area)^18^. While the majority of the synthesized compounds were predicted to show good gastrointestinal absorption, none were predicted to cross the blood brain barrier.

**Table 3:** ADME evaluation of **3a**-**o**.

Molecule	MW^**a**^	H-bond acceptors^**b**^	H-bond donors^**c**^	TPSA^**d**^	WLOGP^**e**^	GI Absorption^f^		Lipinski violations^**g**^	PAINS alerts^**h**^
**3a**	383.55	2	3	88.74	5.12		High	1	1
**3b**	411.63	2	3	88.74	5.73		High	1	1
**3c**	413.58	3	3	97.97	5.13		High	0	1
**3d**	397.58	2	3	88.74	5.43		High	1	1
**3e**	397.58	2	3	88.74	4.83		High	0	1
**3f**	432.02	2	3	88.74	5.49		High	1	1
**3g**	401.54	3	3	88.74	5.68		High	1	1
**3h**	389.63	2	3	88.74	5.12		High	0	1
**3i**	462.45	2	3	88.74	5.88		High	1	1
**3j**	452.44	2	3	88.74	6.42		Low	1	1
**3k**	397.58	2	3	88.74	5.43		High	1	1
**3l**	418.34	2	3	88.74	5.77		High	1	1
**3m**	401.54	3	3	88.74	5.68		High	1	1
**3n**	425.63	2	3	88.74	6.24		Low	1	1
**3o**	411.6	2	3	88.74	5.03		High	1	1

^a^Molecular weight.

^b^Hydrogen bond acceptor.

^c^Hydrogen bond donor.

^d^Total polar surface area.

^e^Logarithm of partition coefficient between n-octanol and water.

^f^Gastrointestinal absorption.

^g^Violations of Lipinski’s rule of five.

^h^PAINS (Pan-assay interference compounds) alert.

Synthesized compounds’ codes were written in bold text.

The results of the SwissADME analysis further indicated that all compounds satisfy Lipinski’s rule of five^19^. Druglikeness was also evaluated through applying a pan-assay interference compound (PAINS) filter^20^, with all synthesized compounds found to not share any significant similarity with known PAINS. Thus, these compounds could be further optimized and considered as viable drug candidates.

## Conclusion

A series of thiosemicarbazones with phenolic moieties appended to the thiosemicarbazone backbone were synthesized with the aim of generating a chemical scaffold with potent, selective aldose reductase (ALR2) inhibitory activity as well as antioxidant activity. Such a scaffold could potentially possess a synergistic ability to treat diabetic complications through dual aldose reductase inhibition and oxidative stress suppression. In the current study, an *in vitro* ALR2 inhibition assay demonstrated that compounds **3f**, **3g**, **3j**, **3l** and **3m** are strong and selective ALR2 inhibitors with IC_50_ values in the low micromolar range (3.12, 2.38, 4.1, 1.46 and 1.18 µM, respectively). **3m**, the most potent inhibitor of the set, showed strong antioxidant properties with a percent free radical scavenging activity of 75.95%. Molecular docking and molecular dynamics simulation studies were used to suggest a molecular-scale rationale for the selective ALR2 inhibitory activity of **3m**. This compound therefore represents a potential drug candidate for treatment of diabetic complications.

## Experimental

### Materials and methods

The AKR1B1 expression plasmid (pDONR223_AKR1B1_WT) was a gift from Jesse Boehm, Matthew Meyerson and David Root (Addgene plasmid # 82928; http://n2t.net/addgene:82928; RRID: Addgene_82928). Biological assay substrates (D,L-glyceraldehyde and sodium-D-glucoronate) and nicotinamide adenine dinucleotide phosphate (NADPH) were purchased from Sigma Aldrich (Merck KGaA, USA). Synthetic building blocks, reagents, solvents and thin layer chromatography plates were purchased from Sigma Aldrich. Fourier-transform infrared spectroscopy (FTIR) analysis in the range of 4000–500 cm^−1^ was performed using a Bruker Vector-22 spectrometer. All NMR spectra were obtained at room temperature using a Bruker Ascend 400 MHz NMR spectrometer and interpreted using ACD/NMR Processor Academic Edition software; chemical shifts (δ H) are expressed in parts per million (ppm) relative to deuterated chloroform (CDCl_3_; residual signal ^1^H δ = 7.26, ^13^C δ = 77.2) or deuterated dimethyl sulfoxide (DMSO-d_6_; residual signal ^1^H δ = 2.50, ^13^C δ = 39.5), coupling constants are expressed in Hz and multiplicities in ^1^H NMR spectra are quoted as follows: s = singlet, d = doublet, t = triplet q = quartet, dd = doublet of doublets, m = multiplet. Analytical liquid chromatography–mass spectrometry (LC-MS) was employed to monitor reaction progression and for compound identification. LC-MS analysis was performed on an Agilent InfinityLab LC/MSD System consisting of an Agilent 1290 Infinity II Analytical-Scale LC Purification System coupled to a 6120 Quadrupole mass spectrometer. High-performance liquid chromatography was carried out using an Onyx™ Monolithic C18 column (50 x 4.6 mm) with water (A) and acetonitrile (B) as the mobile phases, with formic acid (0.1%) added to both to ensure acidic conditions throughout the analysis. Gradient conditions used were as follows: Method A (5 min), flow rate 1.0 mL/min, 100 μL was split via a zero dead volume T piece which passed into the mass spectrometer. The wavelength range of the UV detector was 220–500 nm. Gradient progressed from 95% A/5% B to 10% A/90% B over three minutes, then to 5% A/95% B over a further 30 seconds, was held constant at 5% A/95% B for a further minute and finally returned to 95% A/5% B over a final 30 seconds. Method B (10 min), flow rate 0.5 mL/min, 200 μL was split via a zero dead volume T piece which passed into the mass spectrometer. The wavelength range of the UV detector was 220–400 nm. Gradient progressed from 95% A/5% B to 50% A/50% B over three minutes, then to 20% A/80% B over two further minutes, to 5% A/95% B over a further 1.5 minutes, was held constant at 5% A/95% B for a further 1.5 minutes, returned to 95% A/5% B over a further 0.2 minutes and remained at 95% A/5% B for a further 1.8 minutes. Analytical liquid chromatography was carried out using the following parameters: injection volume 10 μL; draw speed 100 μL/min; ejection speed 400 μL/min; wait time after drawing 1.2 s. Mass spectrometry data (both ESI+ and ESI- modes) were collected using the following parameters: capillary voltage 4 kV (ESI+), 3.5 kV (ESI-); drying gas flow 13.0 L/min; nebulizer pressure 50 psig (method A), 30 psig (method B), 60 psig (maximum); drying gas temperature 350°C; mass range 150–1,200 Da; fragmentor 70; gain 1.00; stepsize 0.10; speed 2,600 u/sec. High resolution mass spectra (HRMS) were obtained on a Thermo Navigator mass spectrometer coupled with liquid chromatography (LC) using electrospray ionisation (ES) and time-of-flight (ToF) mass spectrometry.

### Enzyme inhibition assay

For the determination of enzyme activity of ALR1 and ALR2, 100 µL of reaction mixture was composed of 20 µL of buffer (100 mM sodium phosphate, pH 6.2), 30 µL of enzyme extract, 20 µL of substrate (10 mM), 20 µL of cofactor (0.1 mM of NADPH) and 10 µL of test compound (1 mM)^6,21^. The reaction mixture without cofactor was incubated at 32°C for 10 min, then the enzymatic reaction was initiated with the addition of NADPH and monitored for 5 minutes. Similar protocols were followed for ALR1 and ALR2, however the substrate was different for each enzyme, with sodium-D-glucoronate and DL-glyceraldehyde used as the substrates for the ALR1 and ALR2 assays, respectively. Sorbinil was employed as standard inhibitor of ALR2 while valproic acid was used as a standard inhibitor for ALR1. In addition, similar protocols were adopted for human AKR1B1 that was expressed in *E. coli* BL21 (DE3), though the determined protein concentration was 12 µg/mL for the expressed enzyme. Detailed protocols for the preparation of ALR1, ALR2 and expressed human AKR1B1 can be found in the supporting information.

The newly synthesized N-substitute thiosemicarbazones (**3a**-**o**) were dissolved in 100% DMSO and diluted with deionized water, keeping the DMSO concentration equal to 0.1% in the assay. Compounds were initially tested for percent inhibition at a concentration of 100 µM and IC_50_ values were determined for various dilutions up to 10 nM. Where compounds showed percent inhibition greater than 50%, their IC_50_ values were calculated through non-linear regression analysis using GraphPad Prism version 8.

### Methodology for docking and simulation studies

To investigate the probable binding mode of the specific inhibitor **3m**, molecular docking and molecular dynamics simulation studies were performed. The FlexX utility of BioSolveIT’s LeadIT software package was used to perform the docking studies^22^. The x-ray crystallographic structure of ALR2 (PDB ID 3FX4) was downloaded and prepared using the default docking parameters of the software^23^. Docking was performed in the presence of cofactor NADP. Initially the docking protocol was revalidated by redocking the co-crystallized ligand and comparing its RMSD value. Enthalpy entropy hybrid approach of FlexX utility was used for scoring and ranking of the conformational poses. The highest scoring poses were further subjected to HYDE assessment in order to assess their binding affinities^24,25^.

Molecular dynamic simulation of **3m** was carried out using GROMACS^26,27^. The latest CHARMM36 forcefield was used with TIP3P as an explicit water model^28^. The docked pose of **3m** was used as an initial coordinate and the topology and parameter files were obtained using CHARMM General Force Field (CGENFF) web-based server (https://cgenff.umaryland.edu). The protein–cofactor–inhibitor complex was prepared and wrapped in the TIP3P water box and neutralized with Na^+^ and Cl^-^ counterions. The complex system was minimized using steepest decent and conjugate gradient methods until the maximum force experienced by the system was less than 10^3^ KJ mol^−1^ nm^−1^. The system was allowed to equilibrate for 100 ps using NVT (isothermal-isochoric) and NPT (isothermal-isobaric) ensemble. The complex system was observed to reach 300 K temperature and the pressure was observed to be around 1 atmosphere prior to running the production run. An MD simulation of about 50 ns was performed. Twin-range van der Waals and coulomb interactions were used to determine the non-bonded interactions with a cutoff of 1.0 nm. VMD v9.13 and XMGRACE v5.1.19 were used for visualization and plotting of graphs^29,30^.

### General procedure for synthesis of thiosemicarbazone derivatives (3a-o)

The thiosemicarbazone derivatives (**3a-o**) were synthesized by adding equimolar quantities (1 mmol) of the appropriate N^4^-substituted thiosemicarbazide **(1)** and 3,5-di-tert-butyl-2-hydroxybenzaldehyde **(2)** to an oven dried flask, dissolving in 10 mL methanol and adding a few drops of glacial acetic acid as a catalyst^7,31,32^. The reaction mixture was then heated under reflux for 2–3 hours until the reaction was complete as shown by TLC. The mixture was then cooled to room temperature, allowing the product thiosemicarbazone to precipitate. The crude product was filtered under vacuum, washed with hot methanol followed by ether and then oven dried. Finally, the crude product was recrystallized from ethanol to afford the target thiosemicarbazone in good to excellent yield (79–90%).Characterization data for synthesized thiosemicarbazone derivatives is provided below.

#### 2-(3,5-di-tert-butyl-2-hydroxybenzylidene)-N-phenylhydrazinecarbothioamide (3a)

White solid, yield 81%, m.p. 178–180 °C, IR ʋ_max_ (cm^−1^) 1190 (C=S), 1580 (C=N), 3245, 3310 (N-H), 3412 (OH), ^1^H NMR (DMSO-*d*_*6*_) δ ppm; 1.28 (s, 9H, tert-butyl), 1.40 (s, 9H, tert-butyl), 7.18–7.23 (m, 2H, Ar-H), 7.30 (s, 1H, Ar-H), 7.37 (t, 2H, Ar-H, *J* = 7.5 Hz), 7.48 (s, 1H, Ar-H), 7.51 (s, 1H, Ar-H), 8.37 (s, 1H, N=CH), 9.9 (s, 1H, NH-CS), 10.17 (s, 1H, NH-N), 11.69 (s,1H, OH), ^13^C NMR (DMSO-*d*_*6*_) δ ppm; 29.50, 31.39 (CH_3_ of tertiary butyl moiety), 34.00, 34.77 (C of tertiary butyl moiety), 117.92, 125.33, 125.81, 125.99, 128.30, 136.13, 139.44, 140.98, 148.32, 153.40 (Ar-C), 148.32 (CH=N), 176.39 (C=S), Anal calcd for C_22_H_29_N_3_OS (383.55); C, 68.89; H, 7.62; N, 10.96, Found C, 68.97; H, 7.68; N, 10.88. LC-ESI-MS, *m/z* (%): 384.20 [M+H]^+^ (100).

#### 2-(3,5-di-tert-butyl-2-hydroxybenzylidene)-N-(2,4-dimethylphenyl)hydrazine carbothioamide (3b)

Pale yellow solid, yield 86%, m.p. 186–188 °C, IR ʋ_max_ (cm^−1^) 1215 (C=S), 1602 (C=N), 3228, 3317 (N-H), 3398 (OH), ^1^H NMR (CDCl_3_) δ ppm; 1.34 (s, 9H, tert-butyl), 1.44 (s, 9H, tert-butyl), 2.34 (s, 3H, CH_3_), 2.37 (s, 3H, CH_3_), 7.08–7.14 (m, 3H, Ar-H), 7.42–7.46 (m, 2H, Ar-H), 8.13 (s, 1H, N=CH), 8.17 (s, 1H, NH-CS), 9.99 (s, 1H, NH-N), 10.48 (s,1H, OH), ^13^C NMR (DMSO-*d*_*6*_) δ ppm; 17.82 (CH_3_), 20.71 (CH_3_), 29.49, 31.38 (CH_3_ of tertiary butyl moiety), 33.99, 34.77 (C of tertiary butyl moiety), 118.04, 125.68, 125.85, 126.66, 128.49, 130.81, 135.25, 135.99, 136.14, 140.99, 153.25 (Ar-C), 147.93 (CH=N), 177.16 (C=S), Anal calcd for C_24_H_33_N_3_OS (411.60); C, 70.03; H, 8.08; N, 10.21, Found C, 70.11; H, 8.01; N, 10.29. LC-ESI-MS, *m/z* (%): 413.00 [M+H]^+^ (100).

#### 2-(3,5-di-tert-butyl-2-hydroxybenzylidene)-N-(3-methoxyphenyl)hydrazine carbothioamide (3c)

White solid, yield 80%, m.p. 173–175 °C, IR ʋ_max_ (cm^−1^) 1206 (C=S), 1595 (C=N), 3288, 3305 (N-H), 3450 (OH), ^1^H NMR (DMSO-*d*_*6*_) δ ppm; 1.28 (s, 9H, tert-butyl), 1.40 (s, 9H, tert-butyl), 3.76 (s, 3H, OCH_3_), 6.79 (dd, 1H, Ar-H, *J* = 2.1 Hz, 7.8 Hz), 7.08 (dd, 1H, Ar-H, *J* = 2.1 Hz, 7.8 Hz), 7.17–7.20 (m, 2H, Ar-H), 7.27 (s, 1H, Ar-H), 7.23–7.31 (m, 1H, Ar-H), 8.37 (s, 1H, N=CH), 10.13 (s, 2H, NH-CS & NH-N), 10.69 (s,1H, OH), ^13^C NMR (DMSO-*d*_*6*_) δ ppm; 29.50, 31.38 (CH_3_ of tertiary butyl moiety), 33.99, 34.77 (C of tertiary butyl moiety), 55.22 (OCH_3_), 110.73, 111.52, 117.55, 117.91, 125.81, 125.96, 129.04, 136.13, 140.53, 140.96, 153.43, 159.21 (Ar-C), 148.34 (CH=N), 175.99 (C=S), Anal calcd for C_23_H_31_N_3_O_2_S (413.58); C, 66.79; H, 7.56; N, 10.16, Found C, 66.87; H, 7.48; N, 10.24. LC-ESI-MS, *m/z* (%): 414.50 [M+H]^+^ (100).

#### 2-(3,5-di-tert-butyl-2-hydroxybenzylidene)-N-(o-tolyl)hydrazinecarbothioamide (3d)

Yellow solid, yield 85%, m.p. 182–184 °C, IR ʋ_max_ (cm^−1^) 1218 (C=S), 1615 (C=N), 3290 (N-H), 3427 (OH), ^1^H NMR (CDCl_3_) δ ppm; 1.33 (s, 9H, tert-butyl), 1.45 (s, 9H, tert-butyl), 2.40 (s, 3H, CH_3_), 7.08 (d, 1H, Ar-H, *J* = 3.2 Hz), 7.24 (s, 1H, Ar-H), 7.27 (s, 1H, Ar-H), 7.28 (s, 1H, Ar-H), 7.43–7.47 (m, 2H, Ar-H), 8.09 (s, 1H, N=CH), 8.31 (s, 1H, NH-CS), 9.87 (s, 2H, NH-N & OH), ^13^C NMR (DMSO-*d*_*6*_) δ ppm; 20.65 (CH_3_), 29.50, 31.38 (CH_3_ of tertiary butyl moiety), 33.99, 34.77 (C of tertiary butyl moiety), 117.92, 125.77, 125.97, 128.70, 128.78, 134.12, 134.56, 136.11, 136.67, 136.83, 140.96, 153.36 (Ar-C), 148.20 (CH=N), 176.61 (C=S), Anal calcd for C_23_H_31_N_3_OS (397.58); C, 69.48; H, 7.86; N, 10.57, Found C, 69.57; H, 7.80; N, 10.68. LC-ESI-MS, *m/z* (%): 398.60 [M+H]^+^ (100).

#### N-benzyl-2-(3,5-di-tert-butyl-2-hydroxybenzylidene)hydrazinecarbothioamide (3e)

White solid, yield 80%, m.p. 190–192 °C, IR ʋ_max_ (cm^−1^) 1236 (C=S), 1547 (C=N), 3282 (N-H), 3432 (OH), ^1^H NMR (DMSO-*d*_*6*_) δ ppm; 1.26 (s, 9H, tert-butyl), 1.40 (s, 9H, tert-butyl), 4.82 (d, 2H, CH_2_, *J* = 4.5 Hz), 7.14 (d, 1H, Ar-H, *J* = 1.8Hz), 7.30 (d, 1H, Ar-H, *J* = 1.5 Hz), 7.35–7.40 (m, 5H, Ar-H), 8.32 (s, 1H, N=CH), 9.01 (s, 1H, NH-CS), 9.79 (s, 1H, NH-N), 11.47 (s,1H, OH), ^13^C NMR (DMSO-*d*_*6*_) δ ppm; 29.50, 31.36 (CH_3_ of tertiary butyl moiety), 33.97, 34.75 (C of tertiary butyl moiety), 47.07 (CH_2_), 117.82, 125.74, 126.00, 126.81, 127.20, 128.25, 136.03, 139.43, 141.02, 153.17 (Ar-C), 148.08 (CH=N), 177.35 (C=S), Anal calcd for C_23_H_31_N_3_OS (397.58); C, 69.48; H, 7.86; N, 10.57, Found C, 69.39; H, 7.95; N, 10.66. LC-ESI-MS, *m/z* (%): 398.60 [M+H]^+^ (100).

#### N-(4-chlorobenzyl)-2-(3,5-di-tert-butyl-2-hydroxybenzylidene)hydrazine carbothioamide (3f)

White solid, yield 88%, m.p. 184–186 °C, IR ʋ_max_ (cm^−1^) 1211 (C=S), 1565 (C=N), 3270, 3312 (N-H), 3427 (OH), ^1^H NMR (DMSO-*d*_*6*_) δ ppm; 1.27 (s, 9H, tert-butyl), 1.40 (s, 9H, tert-butyl), 4.82 (d, 2H, CH_2_, *J* = 4.5 Hz), 7.14 (d, 1H, Ar-H, *J* = 1.8Hz), 7.30 (d, 1H, Ar-H, *J* = 1.5 Hz), 7.35–7.40 (m, 4H, Ar-H), 8.32 (s, 1H, N=CH), 9.01 (s, 1H, NH-CS), 9.79 (s, 1H, NH-N), 11.47 (s,1H, OH), ^13^C NMR (DMSO-*d*_*6*_) δ ppm; 29.40, 31.26 (CH_3_ of tertiary butyl moiety), 33.87, 34.65 (C of tertiary butyl moiety), 46.97 (CH_2_), 117.71, 125.90, 126.70, 127.10, 128.15, 135.92, 139.33, 140.92, 153.07 (Ar-C), 147.98 (CH=N), 177.25 (C=S), Anal calcd for C_23_H_30_ClN_3_OS (432.02); C, 63.94; H, 7.00; N, 9.73, Found C, 63.87; H, 7.10; N, 9.82. LC-ESI-MS, *m/z* (%): 433.20 [M+H]^+^ (100).

#### 2-(3,5-di-tert-butyl-2-hydroxybenzylidene)-N-(4-fluorophenyl)hydrazine carbothioamide (3g)

Yellow solid, yield 80%, m.p. 176–178 °C, IR ʋ_max_ (cm^−1^) 1245 (C=S), 1625 (C=N), 3295, 3320 (N-H), 3431 (OH), ^1^H NMR (DMSO-*d*_*6*_) δ ppm; 1.28 (s, 9H, tert-butyl), 1.40 (s, 9H, tert-butyl), 7.17–7.23 (m, 3H, Ar-H), 7.31 (d, 1H, Ar-H, *J* = 2.4 Hz), 7.46–7.51 (m, 2H, Ar-H), 8.37 (s, 1H, N=CH), 10.15 (s, 2H, NH-CS & NH-N), 11.70 (s,1H, OH), ^13^C NMR (DMSO-*d*_*6*_) δ ppm; 29.49, 31.37 (CH_3_ of tertiary butyl moiety), 34.00, 34.77 (C of tertiary butyl moiety), 114.83, 115.05, 117.88, 125.88, 126.03, 128.10, 135.78, 136.14, 141.02, 153.35, 158.55, 160.96 (Ar-C), 148.47 (CH=N), 179.57 (C=S), Anal calcd for C_27_H_28_FN_3_OS (401.54); C, 65.81; H, 7.03; N, 10.46, Found C, 65.90; H, 7.11; N, 10.55. LC-ESI-MS, *m/z* (%): 402.10 [M+H]^+^ (100).

#### N-cyclohexyl-2-(3,5-di-tert-butyl-2-hydroxybenzylidene)hydrazinecarbothioamide (3h)

Light yellow solid, yield 79%, m.p. 168–170 °C, IR ʋ_max_ (cm^−1^) 1189 (C=S), 1618 (C=N), 3285 (N-H), 3399 (OH), ^1^H NMR (CDCl_3_) δ ppm; 1.50–1.71 (m, 4H, cyclohexyl), 1.32 (s, 9H, tert-butyl), 1.48 (s, 9H, tert-butyl), 1.76–1.82 (m, 4H, cyclohexyl), 2.15 (m, 2H, cyclohexyl), 4.28–4.39 (m, 1H, cyclohexyl), 6.60 (d, 1H, CH_2_, *J* = 3.8 Hz), 7.07 (d, 1H, Ar-H, *J* = 2.4 Hz), 7.42 (d, 1H, NH-CS, *J* = 2.4 Hz), 8.06 (s, 1H, N=CH), 9.75 (s, 1H, NH-N), 9.89 (s,1H, OH), ^13^C NMR (DMSO-*d*_*6*_) δ ppm; 29.52, 31.36 (CH_3_ of tertiary butyl moiety), 34.10, 34.76 (C of tertiary butyl moiety), 24.89 (Cyclohexyl-C), 31.77 (Cyclohexyl-C), 33.96 (Cyclohexyl-C), 53.06 (Cyclohexyl-C), 118.02, 125.50, 125.65, 136.01, 140.86, 153.25 (Ar-C), 147.25 (CH=N), 176.01 (C=S), Anal calcd for C_22_H_35_N_3_OS (389.60); C, 67.82; H, 9.05; N, 10.79, Found C, 67.90; H, 9.12; N, 10.88. LC-ESI-MS, *m/z* (%): 390.50 [M+H]^+^ (100).

#### N-(4-bromophenyl)-2-(3,5-di-tert-butyl-2-hydroxybenzylidene)hydrazine carbothioamide (3i)

White solid, yield 90%, m.p. 184–186 °C, IR ʋ_max_ (cm^−1^) 1209 (C=S), 1613 (C=N), 3218, 3301 (N-H), 3420 (OH), ^1^H NMR (DMSO-*d*_*6*_) δ ppm; 1.27 (s, 9H, tert-butyl), 1.40 (s, 9H, tert-butyl), 7.20 (d, 1H, Ar-H, *J* = 1.5 Hz), 7.31 (d, 1H, Ar-H, *J* = 1.8 Hz), 7.40–7.43 (m, 2H, Ar-H), 7.54 (d, 2H, Ar-H, *J* = 6.6 Hz), 8.37 (s, 1H, N=CH), 9.84 (s, 1H, NH-CS), 11.18 (s, 1H, NH-N), 11.74 (s,1H, OH), ^13^C NMR (DMSO-*d*_*6*_) δ ppm; 29.40, 31.28 (CH_3_ of tertiary butyl moiety), 33.90, 34.68 (C of tertiary butyl moiety), 117.76, 125.84, 125.96, 128.09, 129.21, 136.06, 138.35, 140.93, 153.31 (Ar-C), 148.55 (CH=N), 176.20 (C=S), Anal calcd for C_22_H_28_BrN_3_OS (462.45); C, 57.14; H, 6.10; N, 9.09, Found C, 57.09; H, 6.18; N, 9.16. LC-ESI-MS, *m/z* (%): 463.60 [M+H]^+^ (100).

#### 2-(3,5-di-tert-butyl-2-hydroxybenzylidene)-N-(2,3-dichlorophenyl)hydrazine carbothioamide (3j)

Yellow solid, yield 82%, m.p. 193–195 °C, IR ʋ_max_ (cm^−1^) 1195 (C=S), 1599 (C=N), 3288, 3302 (N-H), 3418 (OH), ^1^H NMR (DMSO-*d*_*6*_) δ ppm; 1.28 (s, 9H, tert-butyl), 1.40 (s, 9H, tert-butyl), 7.24 (d, 1H, Ar-H, *J* = 1.8 Hz), 7.32 (d, 1H, Ar-H, *J* = 1.8 Hz), 7.40 (t, 1H, Ar-H, *J* = 6.0 Hz), 7.57 (dd, 1H, Ar-H, *J* = 0.9 Hz, 6.6 Hz), 7.63 (d, 1H, Ar-H, *J* = 6.0 Hz), 8.39 (s, 1H, N=CH), 9.57 (s, 1H, NH-CS), 11.13 (s, 1H, NH-N), 11.91 (s, 1H, OH), ^13^C NMR (DMSO-*d*_*6*_) δ ppm; 29.51, 31.37 (CH_3_ of tertiary butyl moiety), 34.02, 34.78 (C of tertiary butyl moiety), 117.93, 125.66, 126.04, 127.83, 128.50, 128.96, 131.80, 136.39, 140.81, 154.25 (Ar-C), 148.48 (CH=N), 176.31 (C=S), Anal calcd for C_22_H_27_Cl_2_N_3_OS (452.44); C, 58.40; H, 6.02; N, 9.29, Found C, 58.49; H, 6.08; N, 9.20. LC-ESI-MS, *m/z* (%): 453.70 [M+H]^+^ (100).

#### 2-(3,5-di-tert-butyl-2-hydroxybenzylidene)-N-(p-tolyl)hydrazinecarbothioamide (3k)

Light yellow solid, yield 80%, m.p. 186–188 °C, IR ʋ_max_ (cm^−1^) 1227 (C=S), 1620 (C=N), 3270, 3318 (N-H), 3429 (OH), ^1^H NMR (DMSO-*d*_*6*_) δ ppm; 1.27 (s, 9H, tert-butyl), 1.40 (s, 9H, tert-butyl), 2.30 (s, 3H, CH_3_), 7.16 (s, 1H, Ar-H), 7.18–7.19 (m, 2H, Ar-H), 7.30 (d, 1H, Ar-H, *J* = 1.8 Hz), 7.34 (d, 2H, Ar-H, *J* = 6.0 Hz), 8.36 (s, 1H, N=CH), 9.92 (s, 1H, NH-CS), 10.07 (s, 1H, NH-N), 11.38 (s, 1H, OH), ^13^C NMR (DMSO-*d*_*6*_) δ ppm; 20.55 (CH_3_), 29.40, 31.28 (CH_3_ of tertiary butyl moiety), 33.90, 34.67 (C of tertiary butyl moiety), 117.82, 125.67, 125.88, 128.60, 128.68, 134.47, 136.01, 136.73, 153.26 (Ar-C), 148.10 (CH=N), 176.73 (C=S), Anal calcd for C_23_H_31_N_3_OS (397.58); C, 69.48; H, 7.86; N, 10.57, Found C, 69.41; H, 7.80; N, 10.65. LC-ESI-MS, *m/z* (%): 398.40 [M+H]^+^ (100).

#### N-(4-chlorophenyl)-2-(3,5-di-tert-butyl-2-hydroxybenzylidene)hydrazine carbothioamide (3l)

Yellow solid, yield 86%, m.p. 188–190 °C, IR ʋ_max_ (cm^−1^) 1201 (C=S), 1606 (C=N), 3265, 3317 (N-H), 3405 (OH), ^1^H NMR (DMSO-*d*_*6*_) δ ppm; 1.27 (s, 9H, tert-butyl), 1.40 (s, 9H, tert-butyl), 7.20 (d, 1H, Ar-H, *J* = 1.5 Hz), 7.31 (d, 1H, Ar-H, *J* = 8.0 Hz), 7.40–7.43 (m, 2H, Ar-H), 7.53 (d, 2H, Ar-H, *J* = 6.6 Hz), 8.37 (s, 1H, N=CH), 9.91 (s, 1H, NH-CS), 10.18 (s, 1H, NH-N), 11.74 (s, 1H, OH), ^13^C NMR (DMSO-*d*_*6*_) δ ppm; 29.50, 31.37 (CH_3_ of tertiary butyl moiety), 34.00, 34.77 (C of tertiary butyl moiety), 117.85, 125.94, 126.05, 127.37, 128.19, 129.31, 136.15, 138.44, 141.03, 153.41 (Ar-C), 148.65 (CH=N), 176.30 (C=S), Anal calcd for C_22_H_28_ClN_3_OS (418.00); C, 63.21; H, 6.75; N, 10.05, Found C, 63.29; H, 6.68; N, 10.14. LC-ESI-MS, *m/z* (%): 419.10 [M+H]^+^ (100).

#### 2-(3,5-di-tert-butyl-2-hydroxybenzylidene)-N-(2-fluorophenyl)hydrazine carbothioamide (3m)

White solid, yield 88%, m.p. 180–182 °C, IR ʋ_max_ (cm^−1^) 1182 (C=S), 1622 (C=N), 3299, 3315 (N-H), 3447 (OH), ^1^H NMR (DMSO-*d*_*6*_) δ ppm; 1.28 (s, 9H, tert-butyl), 1.40 (s, 9H, tert-butyl), 7.17–7.22 (m, 3H, Ar-H), 7.31 (d, 1H, Ar-H, *J* = 1.8 Hz), 7.47–7.50 (m, 2H, Ar-H), 8.37 (s, 1H, N=CH), 9.84 (s, 1H, NH-CS), 10.12 (s, 1H, NH-N), 11.67 (s, 1H, OH), ^13^C NMR (DMSO-*d*_*6*_) δ ppm; 29.49, 31.37 (CH_3_ of tertiary butyl moiety), 34.00, 34.77 (C of tertiary butyl moiety), 114.83, 115.05, 117.88, 125.88, 126.03, 128.10, 135.78, 136.14, 141.02, 153.35, 158.55, 160.96 (Ar-C), 148.47 (CH=N), 176.62 (C=S), Anal calcd for C_22_H_28_FN_3_OS (401.54); C, 65.81; H, 7.03; N, 10.46, Found C, 65.90; H, 7.11; N, 10.57. LC-ESI-MS, *m/z* (%): 402.60 [M+H]^+^ (100).

#### 2-(3,5-di-tert-butyl-2-hydroxybenzylidene)-N-(4-isopropylphenyl)hydrazine carbothioamide (3n)

White solid, yield 84%, m.p. 189–191 °C, IR ʋ_max_ (cm^−1^) 1191 (C=S), 1626 (C=N), 3235, 3328 (N-H), 3432 (OH), ^1^H NMR (DMSO-*d*_*6*_) δ ppm; 1.22 (d, 6H, isopropyl CH_3_, *J* = 6.9 Hz), 1.28 (s, 9H, tert-butyl), 1.40 (s, 9H, tert-butyl), 2.83–2.96 (m, 1H, isopropyl CH), 7.19–7.25 (m, 3H, Ar-H), 7.31 (d, 1H, Ar-H, *J* = 1.8 Hz), 7.38 (d, 2H, Ar-H, *J* = 8.1 Hz), 8.37 (s, 1H, N=CH), 10.08 (s, 2H, NH-CS & NH-N), 11.63 (s, 1H, OH), ^13^C NMR (DMSO-*d*_*6*_) δ ppm; 24.04 (CH_3_ of isopropyl moiety), 33.12 (CH of isopropyl moiety), 29.49, 31.38 (CH_3_ of tertiary butyl moiety), 34.09, 34.77 (C of tertiary butyl moiety), 117.94, 125.74, 128.61, 131.25, 136.10, 137.11, 140.94, 145.50, 153.40, 157.98 (Ar-C), 148.17 (CH=N), 176.36 (C=S), Anal calcd for C_25_H_35_N_3_OS (425.63); C, 70.55; H, 8.29; N, 9.87, Found C, 70.49; H, 8.37; N, 9.95. LC-ESI-MS, *m/z* (%): 426.50 [M+H]^+^ (100).

#### 2-(3,5-di-tert-butyl-2-hydroxybenzylidene)-N-phenethylhydrazinecarbothioamide (3o)

Light green solid, yield 81%, m.p. 174–176 °C, IR ʋ_max_ (cm^−1^) 1210 (C=S), 1607 (C=N), 3254, 3311 (N-H), 3421 (OH), ^1^H NMR (DMSO-*d*_*6*_) δ ppm; 1.27 (s, 9H, tert-butyl), 1.42 (s, 9H, tert-butyl), 2.92 (t, 2H, CH_2_, *J* = 5.4 Hz), 3.75 (q, 2H, CH_2_, *J* = 4.8 Hz), 7.13 (d, 1H, Ar-H, *J* = 1.8 Hz), 7.19–7.23 (m, 1H, Ar-H), 7.26–7.33 (m, 5H, Ar-H), 8.28 (s, 1H, N=CH), 8.45 (s, 1H, NH-CS), 9.91 (s, 1H, NH-N), 11.37 (s, 1H, OH), ^13^C NMR (DMSO-*d*_*6*_) δ ppm; 29.87, 31.74 (CH_3_ of tertiary butyl moiety), 34.35, 35.14 (C of tertiary butyl moiety), 35.17 (CH_2_), 45.97 (CH_2_), 114.99, 118.20, 126.05, 126.62, 128.90, 129.10, 136.38, 139.78, 141.38, 153.63 (Ar-C), 148.08 (CH=N), 177.06 (C=S), Anal calcd for C_24_H_33_N_3_OS (411.60); C, 70.03; H, 8.08; N, 10.21, Found C, 70.11; H, 8.01; N, 10.28. LC-ESI-MS, *m/z* (%): 412.70 [M+H]^+^ (100).

## Supplementary Information

Supplementary Information.

## Data Availability

The datasets generated during and/or analysed during the current study are available from the corresponding author on reasonable request.
