# Supplementary material for: Development and exploration of novel substituted thiosemicarbazones as inhibitors of aldose reductase via in vitro analysis and computational study
Source: Sci Rep. 2022 Apr 6;12:5734. doi: 10.1038/s41598-022-09658-z (PMC8986850; doi:10.1038/s41598-022-09658-z)
Supplement: Supplementary file 1 — Supplementary Information. [file 41598_2022_9658_MOESM1_ESM.docx]

**Supporting information**

**Development and Exploration of Novel Substituted Thiosemicarbazones as Inhibitors of Aldose Reductase via In vitro Analysis and Computational Study**

Aqeel Imran^a,b,d^, Muhammad Tariq Shehzad^c^, Syed Jawad Ali Shah^a^, Taha al Adhami^d,^ Mark Laws^d^, Khondaker Miraz Rahman^d^, Rima D. Alharthy^e^, Imtiaz Ali Khan^f^, Zahid Shafiq*^c^, Jamshed Iqbal*^a,b^

^a^Center for Advanced Drug Research, COMSATS University Islamabad, Abbottabad Campus, Abbottabad 22060, Pakistan

^b^Department of Pharmacy, COMSATS University Islamabad, Abbottabad Campus, Abbottabad 22060, Pakistan

^c^Institute of Chemical Sciences, Bahauddin Zakariya University, Multan 60800, Pakistan

^d^School of Cancer and Pharmaceutical Sciences, King’s College London, Franklin-Wilkins Building, 150 Stamford Street, London SE1 9NH, United Kingdom

^e^Chemistry Department, Faculty of Science & Arts, King Abdulaziz University, Rabigh 21911, Saudi Arabia

^f^Department of Entomology, Agricultural University, Peshawar 25130, Khyber Pakhtunkhwa, Pakistan.

**Expression of human AKR1B1 in *E. coli*:**

**Materials**

Expression plasmid (pDONR223_AKR1B1_WT) was a gift from Jesse Boehm & Matthew Meyerson & David Root (Addgene plasmid # 82928; http://n2t.net/addgene:82928 ; RRID:Addgene_82928).

**Expression AKR1B1 in Bacterial system**

The expression plasmid for aldose reductase (pDONR223_AKR1B1_WT) was gift from Addgene in stable culture[1]. Streaking of this culture has been carried out on LB Agar plate then incubated overnight, after growth of colonies , inoculation was performed in LB medium then incubated this medium in shaking incubator at 37ºC. After 48 hours turbidity was measured when OD=0.6 then some of bacterial growth was prepared as 50% glycerol stock. Others are subjected to plasmid isolation following method Miniprep. Meanwhile E. Coli BL21 De3 cells were made competent then transformation with extracted plasmid (AKR1B1) had been carried out heat shock method. Transformed cell ( E.Coli BL21 DE3+AKR1B1) were grown in 37ºC in shaking incubator for 48 hr [2]. The selection for transformed cells was carried out with the help of antibiotic (spectinomycin 50µg/ml). After addition of spectinomycin, non-transformed cells were killed, only transformed cell survived because of spectinomycin resistance gene in the plasmid. Now, E.coli Bl21 De3 have AKR1B1 in its DNA. For the expression of aldose reductase , E.coli BL21DE3 were induced with of IPTG(Isopropyl-d-thiogalactopyranoside)[3]. Now recombinant protein was started to express in the cell. The expressed enzyme was stabilized by addition 10 mM Tris–HCl buffer, 0.5 mM EDTA, 5 mM β-marcaptoethanol at pH 8.0 and 20% (v/v) glycerol. Cells were lysed with lysis buffer and then precipitation with ammonium sulfate up to 80% saturation was done . the expressed enzyme was dialyzed overnight against tris buffer. Protein quantification was performed following Bradford protein assay[4]. Aliqoutes were stored at -80 refrigerator.

**Extraction and isolation of Aldehyde reductase (ALR1).**

The calf’s kidneys were obtained after slaughtering and dissection, dissolved in 3 volumes of sodium phosphate buffer (10mM) which is composed of 2.0 mM EDTA, 2.5mM β-mercaptoethanol, 0.25M solution of sucrose at pH 7.25. First, the homogenisation was carried out then centrifugation of the homogenate was performed at 12000 x g at 0-2^◦^C for 30 minutes. The precipitates containing insoluble lipids were discarded and the collected supernatant layer was treated with 40% saturation of ammonium sulphate. This liquid mixture was subjected to centrifugation at 11000 x g at 0-2^◦^C for 30 minutes. Now, precipitates were again discarded and supernatant was collected and treated with 50% ammonium sulphate and procedure was repeated in the same manner as in previous step. In the last step, the saturation of ammonium sulphate was increased up to 75% and supernatant was subjected to same parameter of centrifugation. At this final step, precipitates were obtained and supernatant layer was discarded. The obtained material of precipitates was composed of ALR1and redissolved in initial buffer that was used during homogenisation. Finally, material was dialyzed overnight with same buffer system. The dialyzed fractions containing ALR1 were stored at -80^◦^C for further use[5].

**Extraction and isolation of Aldose reductase (ALR2).**

The extraction of ALR2 was carried out from calf lenses via adopting method described in

in (Hayman and Kinoshita 1965) with little modification. The lenses were obtained from slaughterhouse immediately after calf’s slaughtering and stored at -20^◦^C until further use. These lense (150-170g) were subjected to homogenization with about three times volume of cold distilled water and then centrifugation was carried out for the homogenate at 10,000 x g for 14 minutes for removal of insoluble lipids particles. Precipitates were discarded and supernatant layer was subjected to 40% saturation of ammonium sulphate and again centrifugation was performed with same parameters for 14 minutes and again precipitates were discarded. For the removal of undesired protein, supernatant was treated with increased 50 % saturation of ammonium sulphate in the similar manners. In the last step, the supernatant was subjected to 75% of saturation with ammonium sulphate to obtain ALR2. Now, the precipitates were collected instead supernatant because precipitated protein was the expected desired ALR2. The precipitated enzyme (ALR2) was redissolved in 50mM solution of sodium chloride and dialyzed for 24 hours against 50mM solution (3.5L) of NaCl. The volume was recorded before and after dialysis, then dialyzed material was treated with liquid nitrogen. The extracted ALR2 was stored in 1mL Eppendorf tube in deep freezer at -80 ^◦^C till further enzymatic studies [6].

**Protocols for Molecular Docking and MD Simulation studies:**

To investigate the probable binding mode of the specific inhibitor **3m** molecular docking and dynamic simulation studies were performed. FlexX utility of BioSolveIT’s LeadIT software was used to perform the docking studies.[7] The x-ray crystallographic structure of ALR2 with PDB ID 3FX4 was downloaded and prepared using the default docking parameters of the software.[8] Docking was performed in the presence of cofactor NADP. Initially the docking protocol was revalidated by redocking the co-crystallized ligand and comparing its RMSD value. Enthalpy entropy hybrid approach of FlexX utility was used for scoring and ranking of the conformational poses. The highest scoring poses were further subjected to HYDE assessment in order to assess their binding affinities.[9, 10]

Molecular dynamic simulation of inhibitor **3m** was carried out using GROMACS.[11, 12] Latest charmm36 forcefield was used with TIP3P as explicit water model.[13] The docked pose of inhibitor **3m** was used as initial coordinate and the topology and parameter files were obtained using Charmm General Force Field (CGENFF) web based server (<https://cgenff.umaryland.edu>). The protein – cofactor – inhibitor complex was prepared and wrapped in TIP3P water box and neutralized with counter Na^+^ and Cl^-^ ions. The complex system was minimized using steepest decent and conjugate gradient method until the maximum force experience by the system was less than 10^3^ KJmol^-1^nm^-1^. The system was allowed to equilibrate for 100ps using NVT (isothermal-isochoric) and NPT (isothermal-isobaric) ensemble. The complex system was observed to reach 300 K temperature and the pressure was observed to be around 1 atmospheric pressure prior to running the production run. MD simulation of about 50ns was performed. Twin-range van der waals and coulombs interactions were used to determine the non-bonded interactions with a cutoff of 1.0nm. VMD v9.13 and XMGRACE v5.1.19 was used for visualization and plotting of graphs. [14, 15]

**IC_50_ graph for expressed ALR2 ( AKR1B1):**


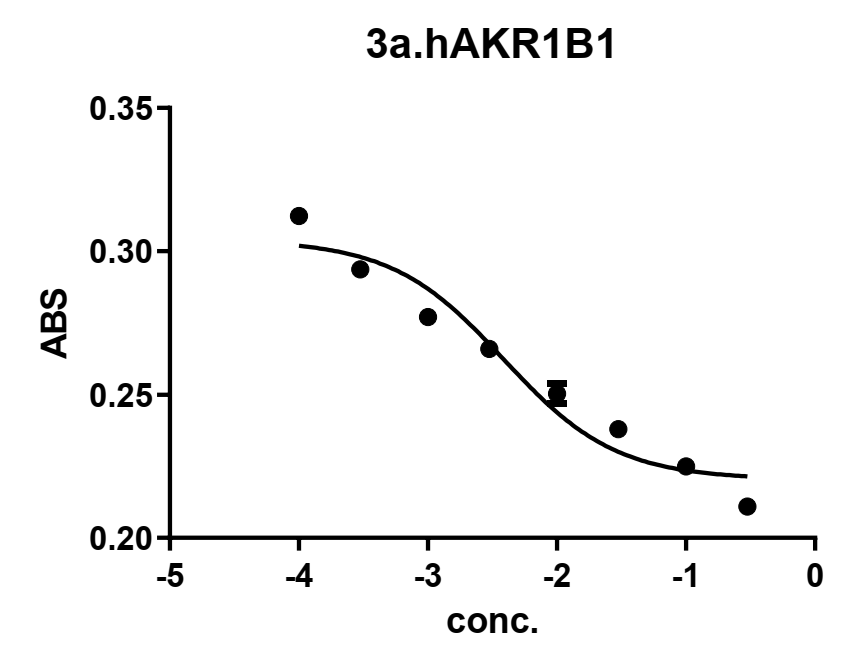

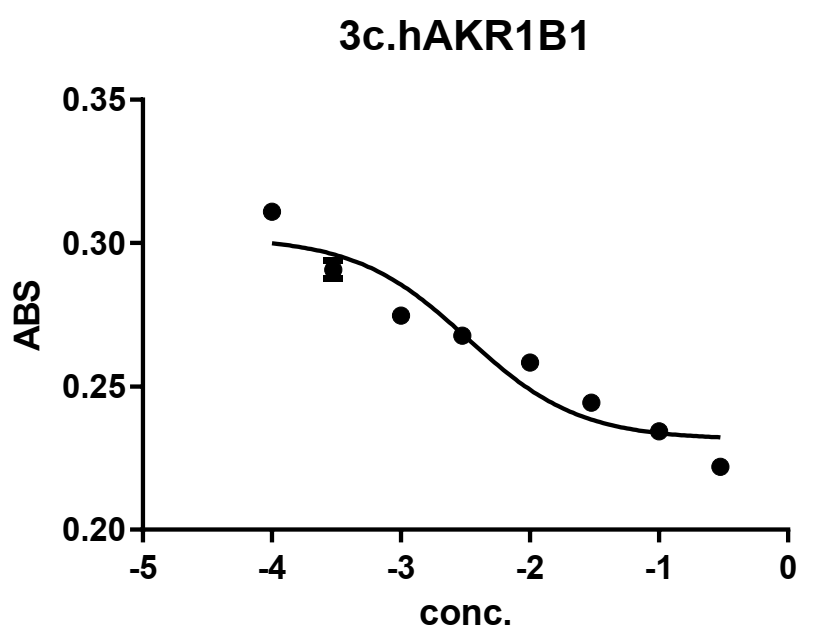


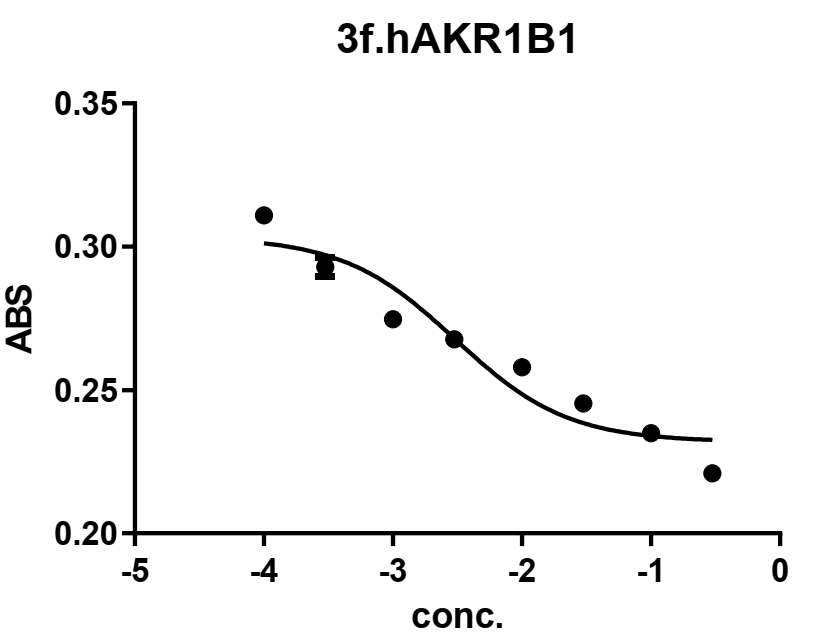

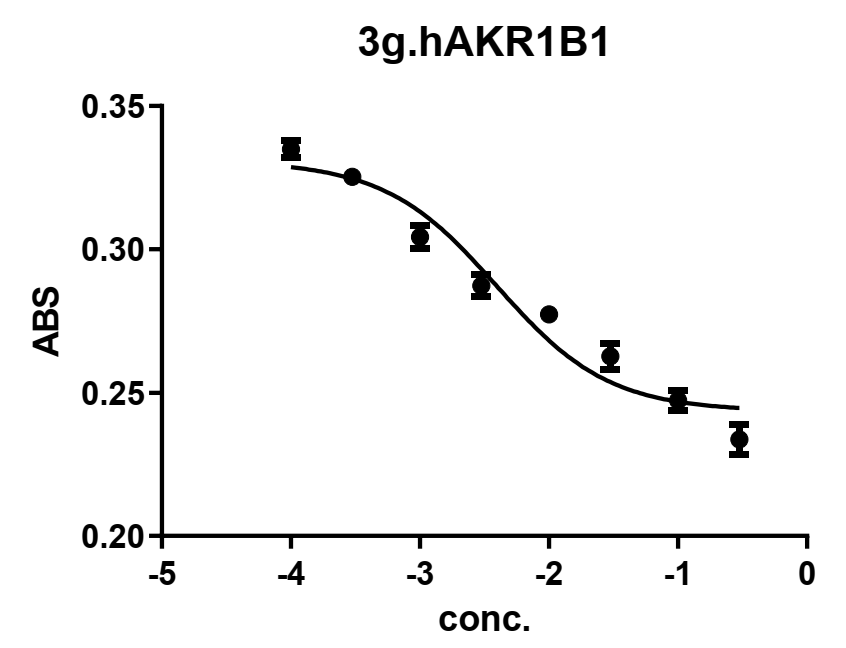


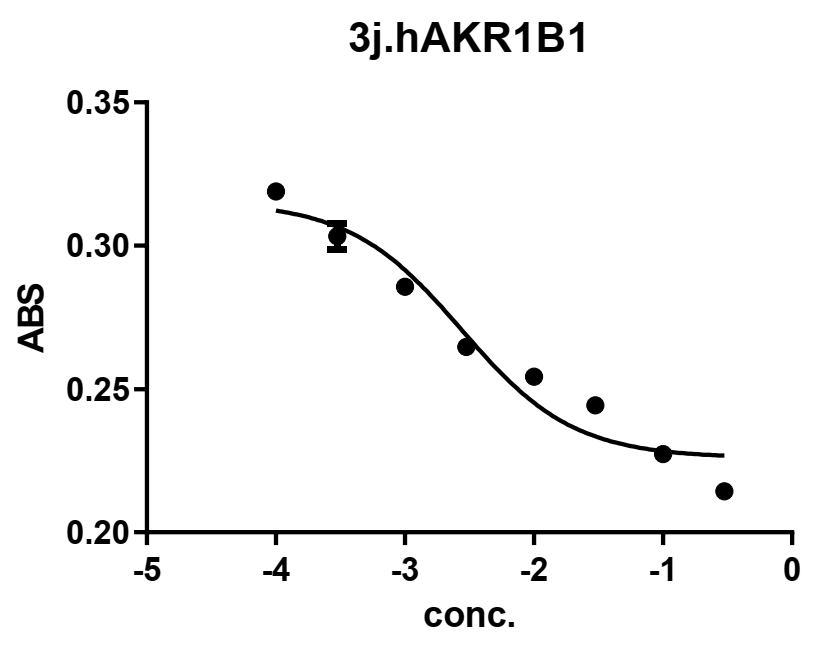

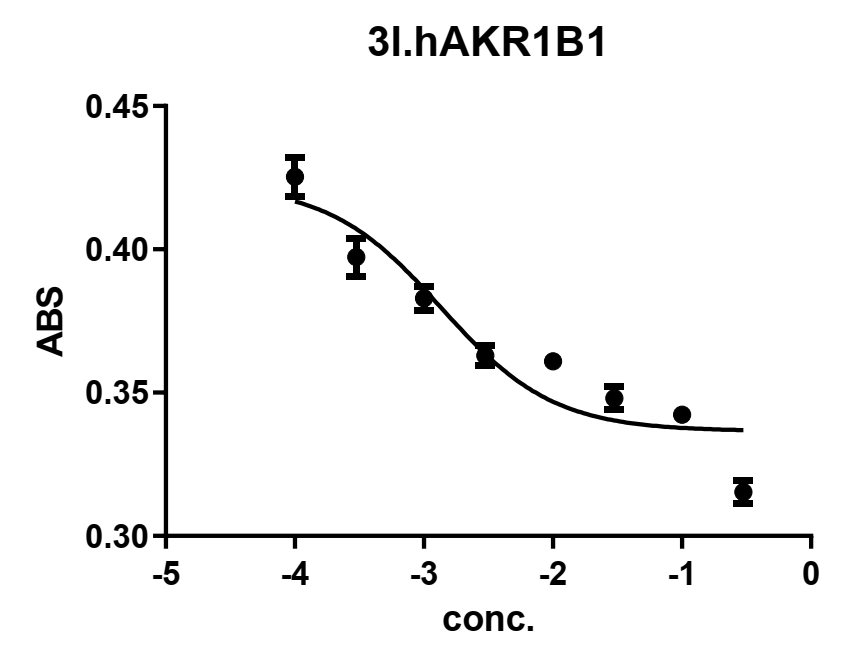


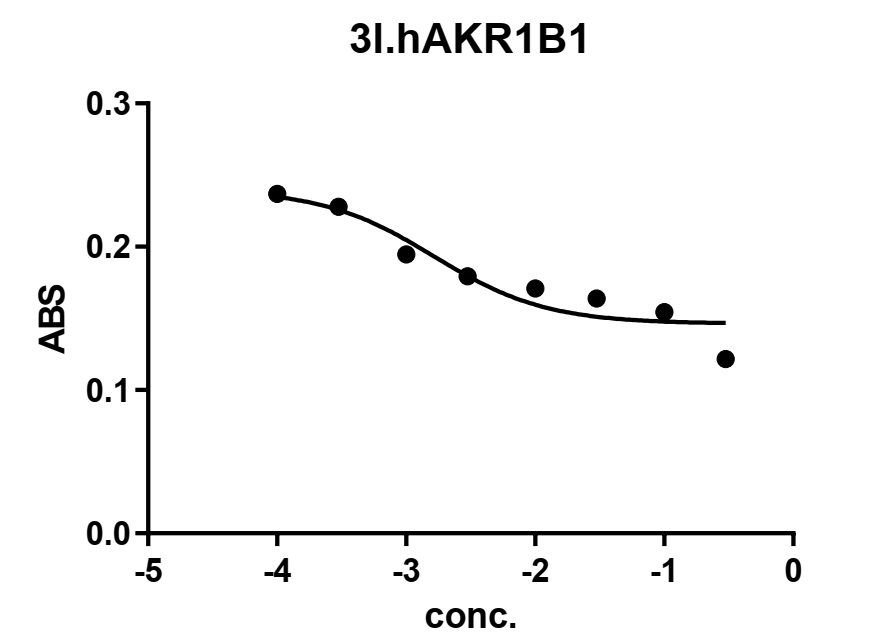


**Supplementary Figure S1:** IC_50_ Graphs of test compounds against ALR2 (AKR1B1)

**Enzyme inhibition of ALR2 eye lenses.**

**Aq1(3a) Aq3 (3c)**


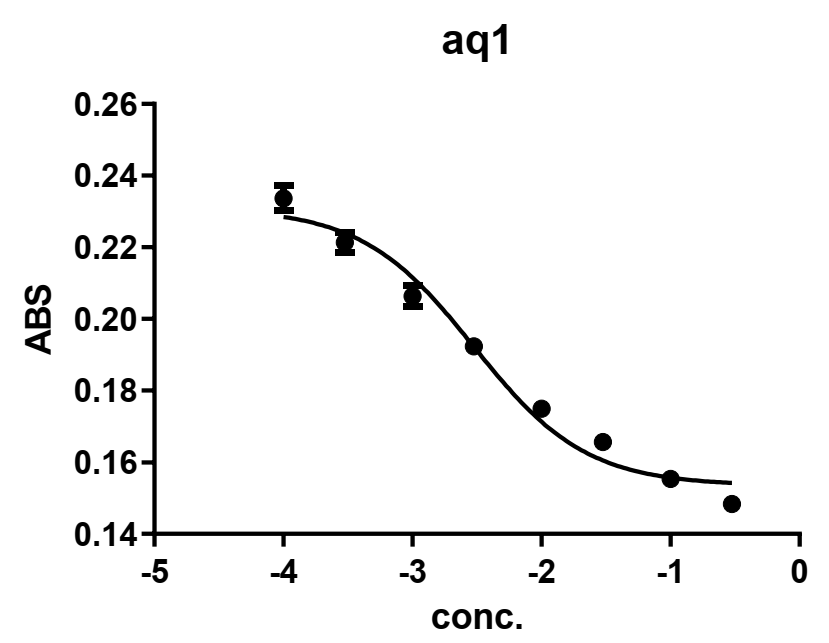

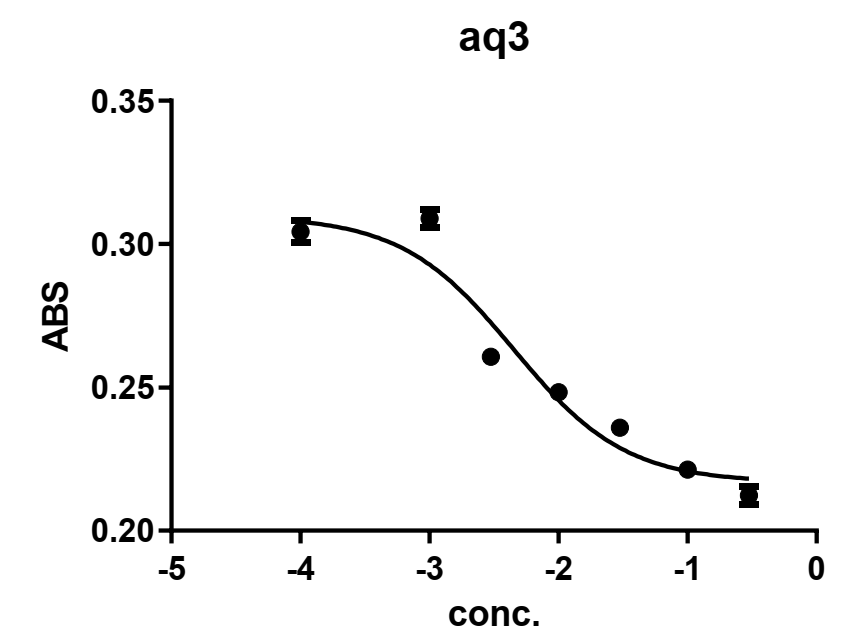


**Aq6 (3f) Aq7 ( 3g)**


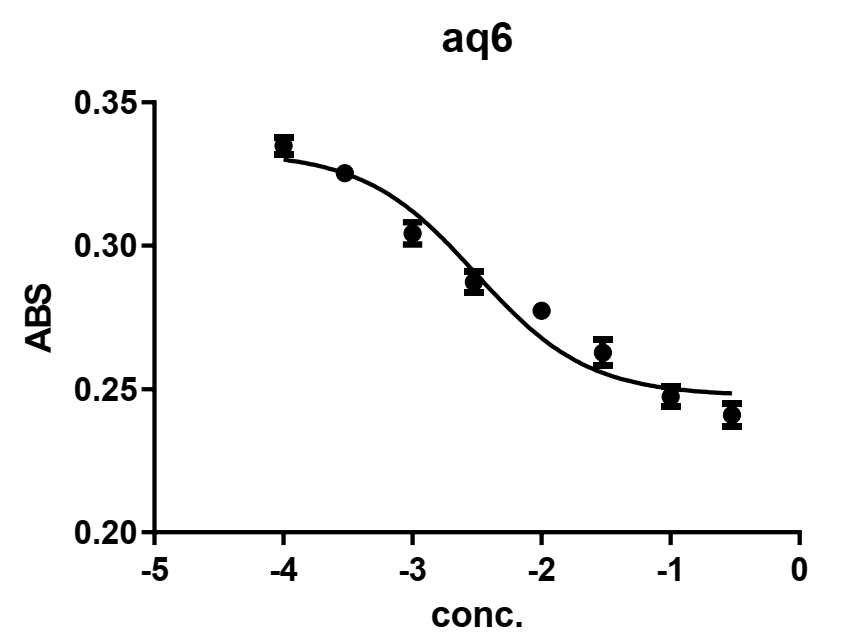

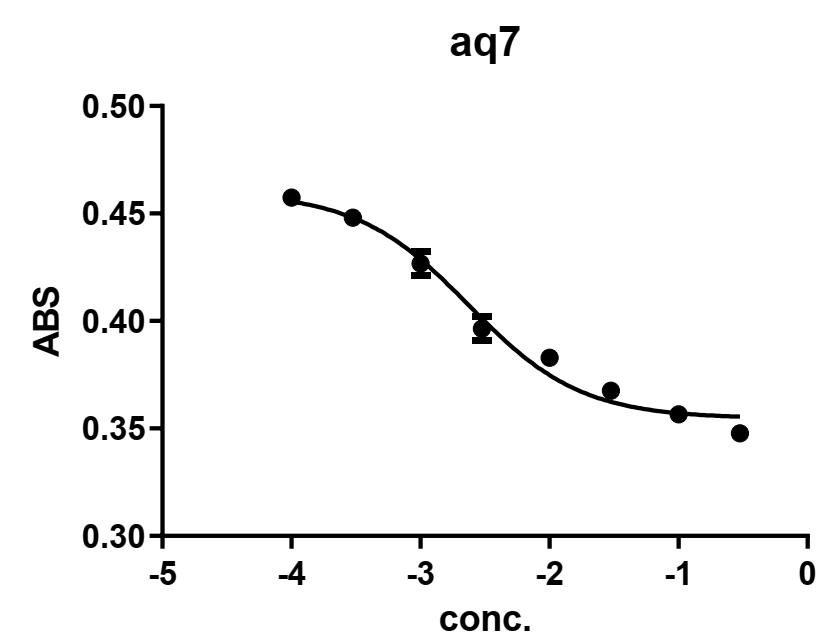


**Aq11 (3j) Aq15 (3l)**


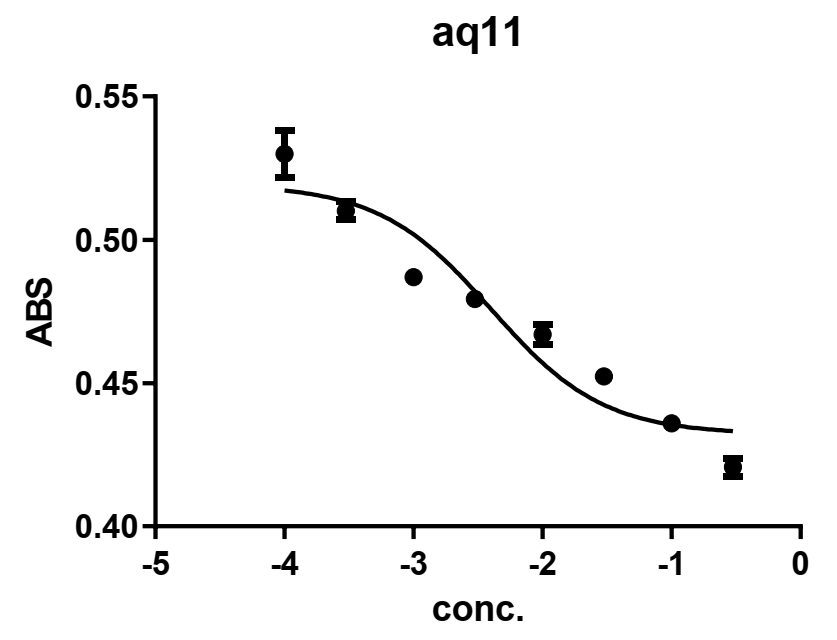

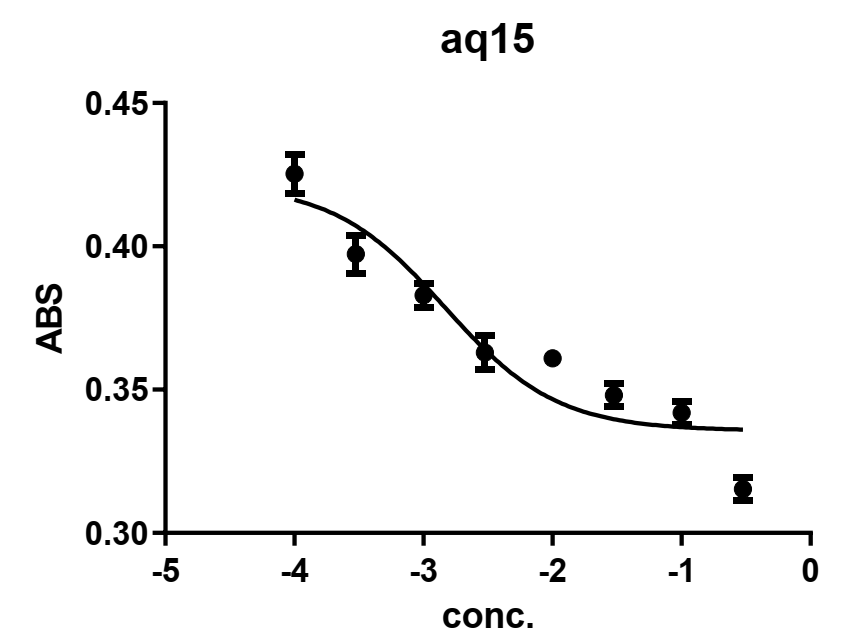


**Aq17 (3m)**


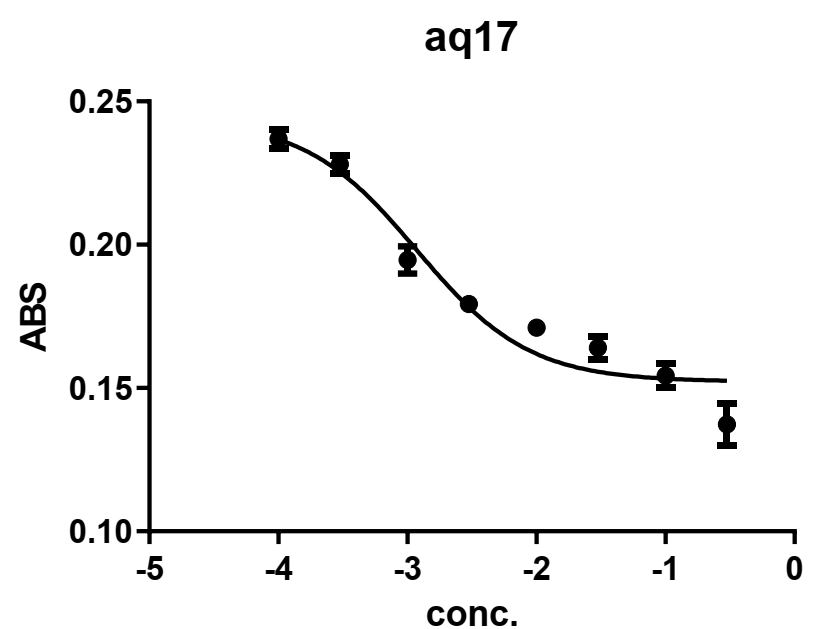


**Supplementary Figure S2:** IC_50_ Graphs of test compounds against ALR2 eye lenses

**IC_50_ graph for ALR1**

**Aq1 ( 3a) Aq3 (3c)**


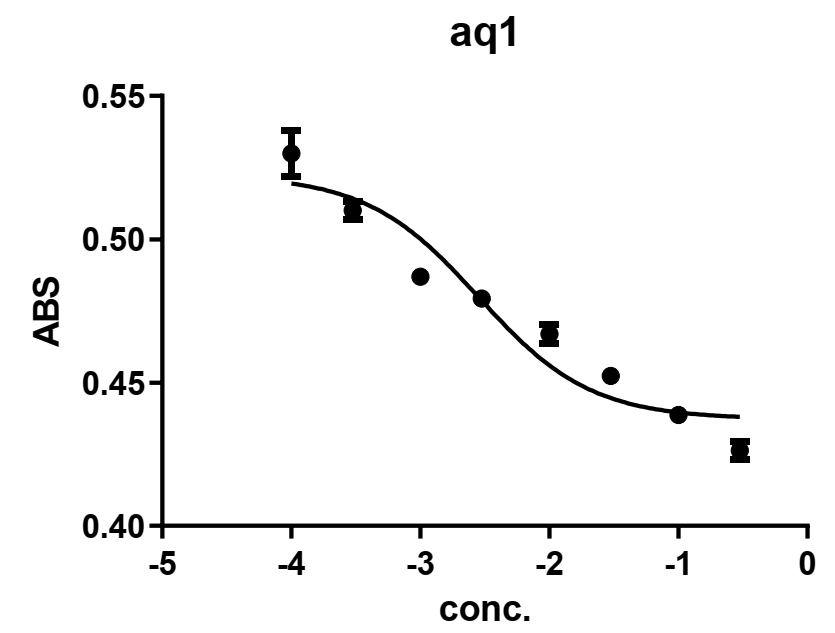

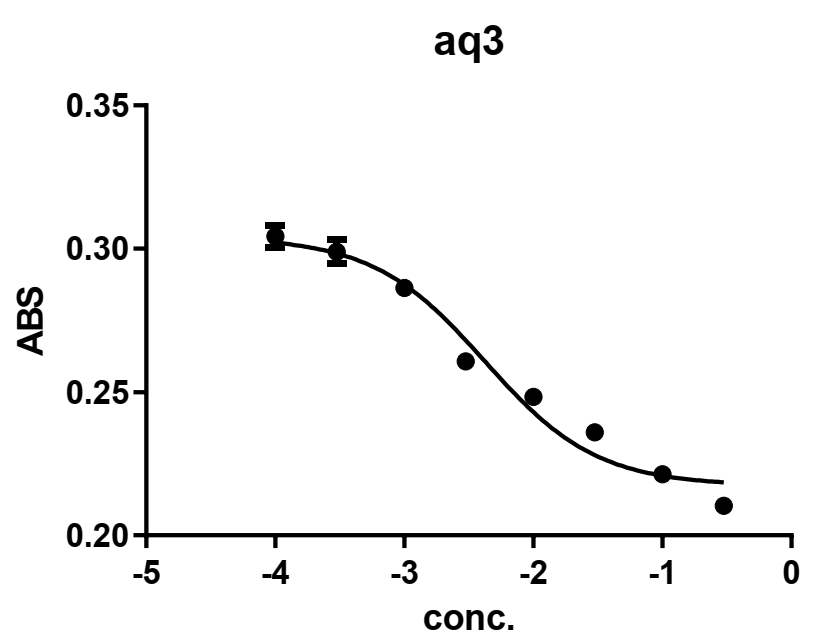


**Supplementary Figure S3:** IC_50_ Graphs of test compounds against ALR1

**References**

[1] Berger, A.H. *et al.* High-throughput phenotyping of lung cancer somatic mutations, *Cancer Cell*, **30**, 214-228 (2016).

[2] Iino, T. *et al.* Tetrahydrobiopterin is synthesized from 6-pyruvoyl-tetrahydropterin by the human aldo-keto reductase AKR1 family members, *Arch. Biochem. Biophys.* **416,** 180-187 (2003).

[3] Pédelacq, J.-D. *et al.* Engineering and characterization of a superfolder green fluorescent protein, *Nat. Biotechnol.* **24**, 79 (2006).

[4] Kruger, N.J. The Bradford method for protein quantitation, The protein protocols handbook, Springer2009, pp. 17-24.

[5] Ward, W.H.*et al.* Ponalrestat: a potent and specific inhibitor of aldose reductase, *Biochem. Pharmacol.* **39,** 337-346 (1990).

[6] Kador, P.F. *et al.* Purified rat lens aldose reductase. Polyol production in vitro and its inhibition by aldose reductase inhibitors, *Biochem. J.* **240,** 233-237 (1986).

[7] Rarey, M., Kramer, B., Lengauer, T. & Klebe, G. A fast flexible docking method using an incremental construction algorithm, *J. Mol. Biol.* **261,** 470-489 (1996).

[8] Carbone, V. *et al.* Structure of aldehyde reductase in ternary complex with a 5-arylidene-2, 4-thiazolidinedione aldose reductase inhibitor, *Eur. J. Med. Chem.* **45,** 1140-1145 (2010).

[9] Reulecke, I., Lange, G., Albrecht, J., Klein, R. & Rarey, M. Towards an integrated description of hydrogen bonding and dehydration: decreasing false positives in virtual screening with the HYDE scoring function, *ChemMedChem.* **3,** 885-897 (2008).

[10] Schneider, N., Lange, G., Hindle, S., Klein, R. & Rarey, M. A consistent description of HYdrogen bond and DEhydration energies in protein–ligand complexes: methods behind the HYDE scoring function, *J. Comput. Aided. Mol. Des.* **27,** 15-29 (2013).

[11] Abraham, M.J. *et al.* GROMACS: High performance molecular simulations through multi-level parallelism from laptops to supercomputers, *SoftwareX.* **1**, 19-25 (2015).

[12] Berendsen, H.J., van der Spoel, D. & van Drunen, R. GROMACS: a message-passing parallel molecular dynamics implementation, *Comput. Phys. Commun.* **91**, 43-56 (1995).

[13] Vanommeslaeghe, K. *et al.* CHARMM general force field: A force field for drug‐like molecules compatible with the CHARMM all‐atom additive biological force fields, *J. Comput. Chem.* **31**, 671-690 (2010).

[14] Turner, P. XMGRACE, 5.1. 19, Center For coastal land-margin research, oregon graduate institute of science technology, Beaverton, Ore, USA (2005).

[15] Humphrey, W, Dalke, A. & Schulten, K. VMD: visual molecular dynamics, *J. Mol. Graph.* **14**, 33-38 (1996).

**^1^H NMR & ^13^C Spectra**

**(3a)**


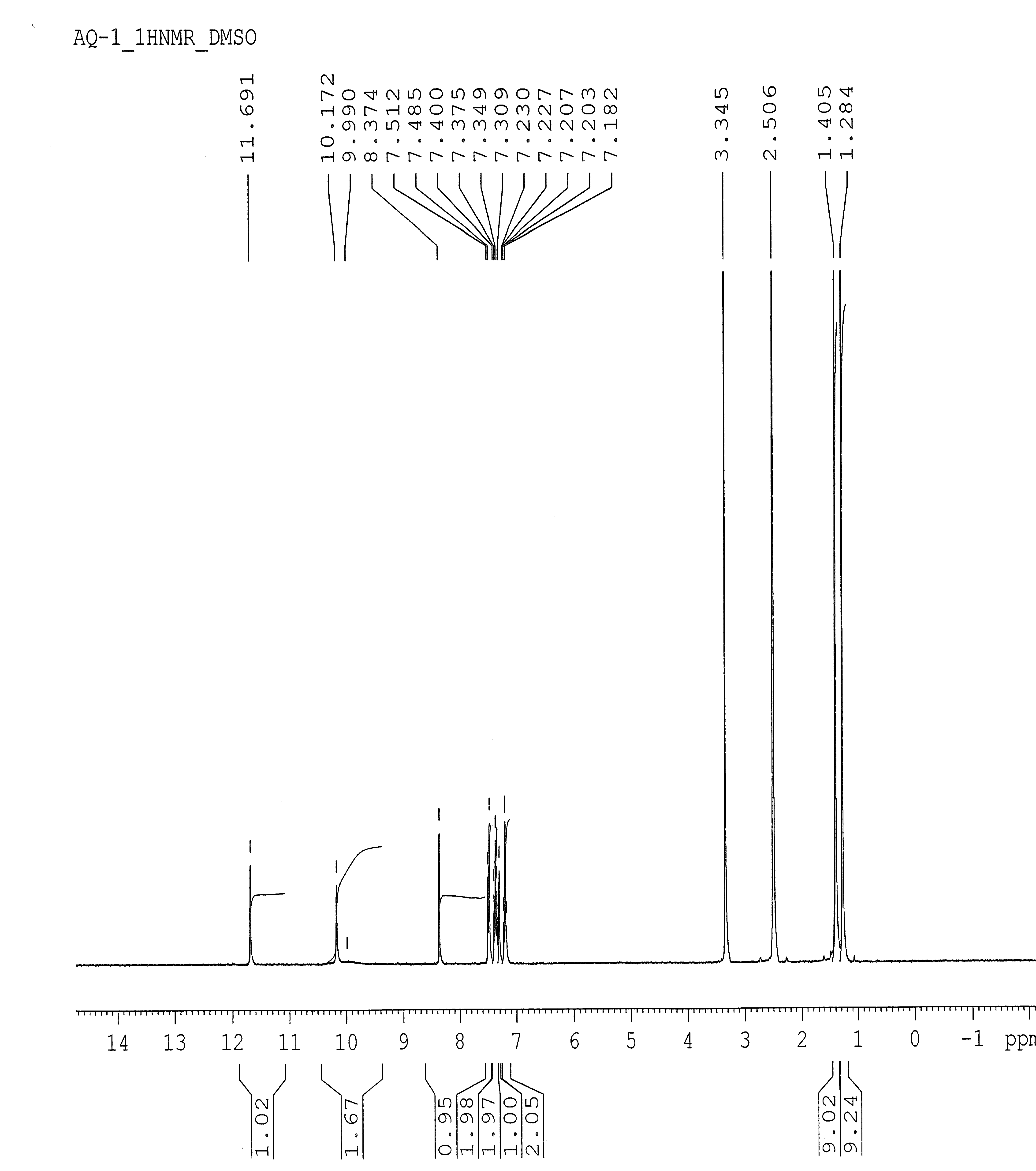


**Supplementary Figure S4:** ^1^H NMR spectra of compound **3a**

**Supplementary Figure S5:** ^13^C NMR spectra of compound **3a**

 **(3b)**


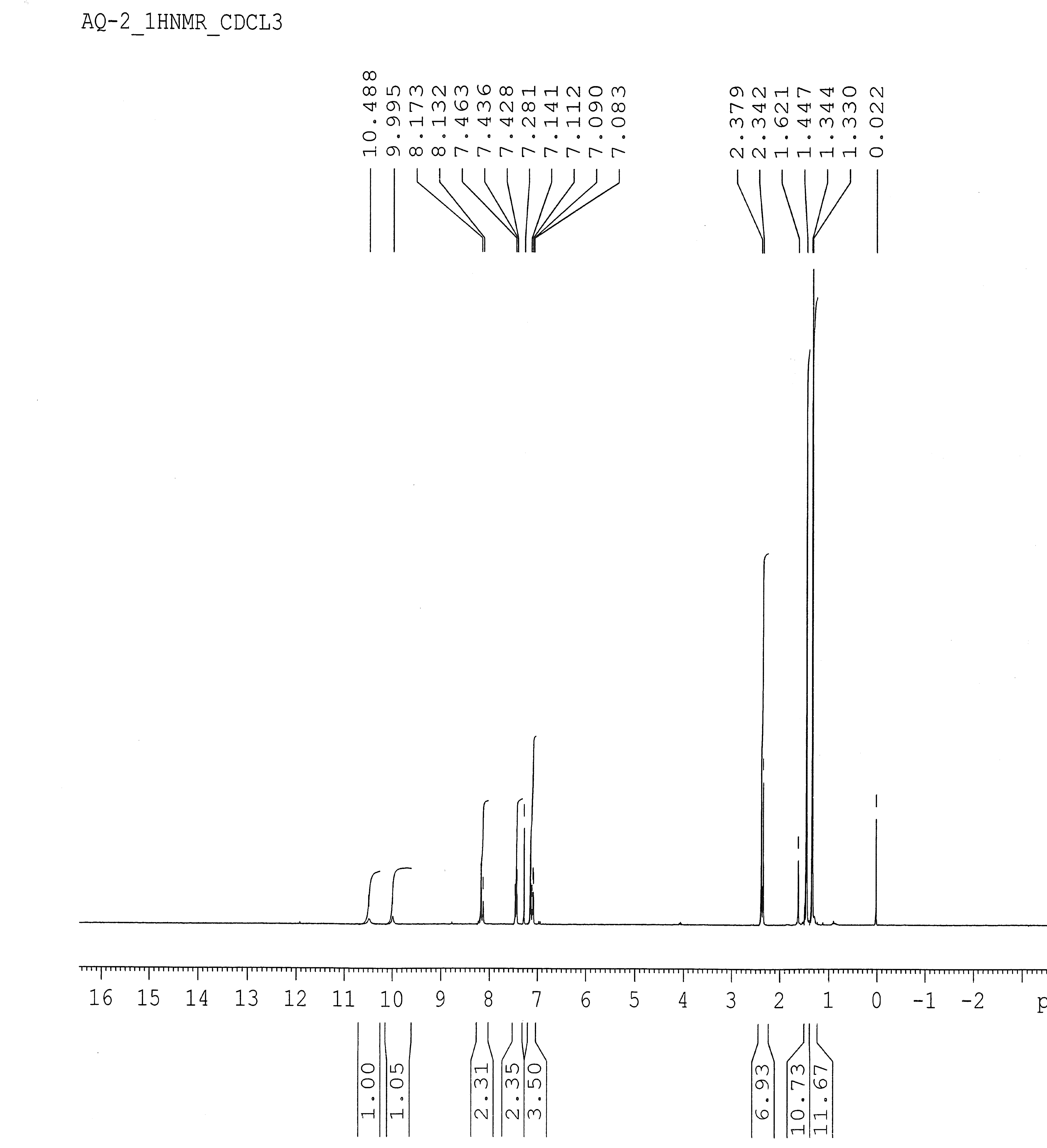


**Supplementary Figure S6:** ^1^H NMR spectra of compound **3b**

**Supplementary Figure S7:** ^13^C NMR spectra of compound **3b**

 **(3c)**


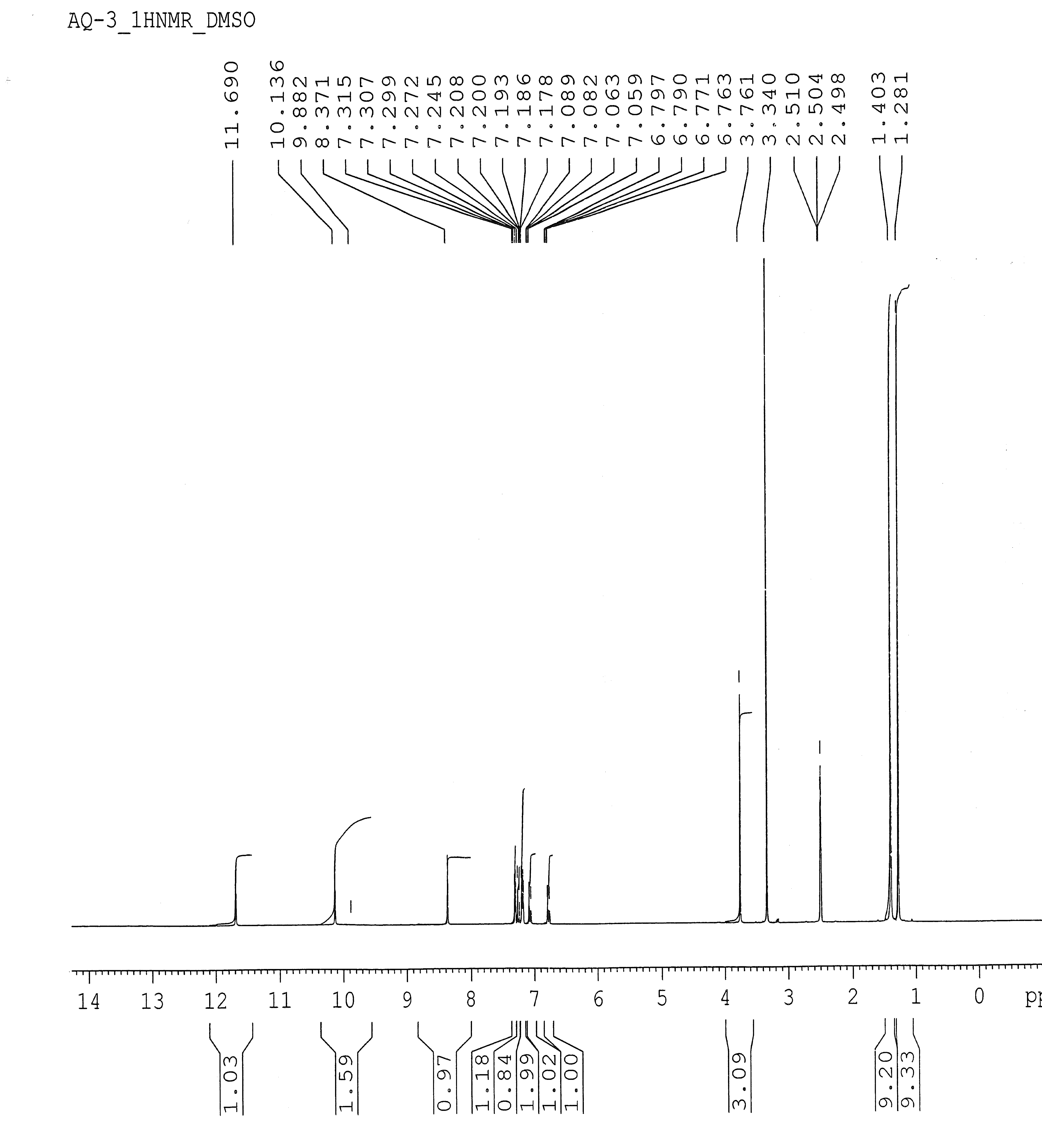


**Supplementary Figure S8:** ^1^H NMR spectra of compound **3c**

**Supplementary Figure S9:** ^13^C NMR spectra of compound **3c**

 **(3d)**


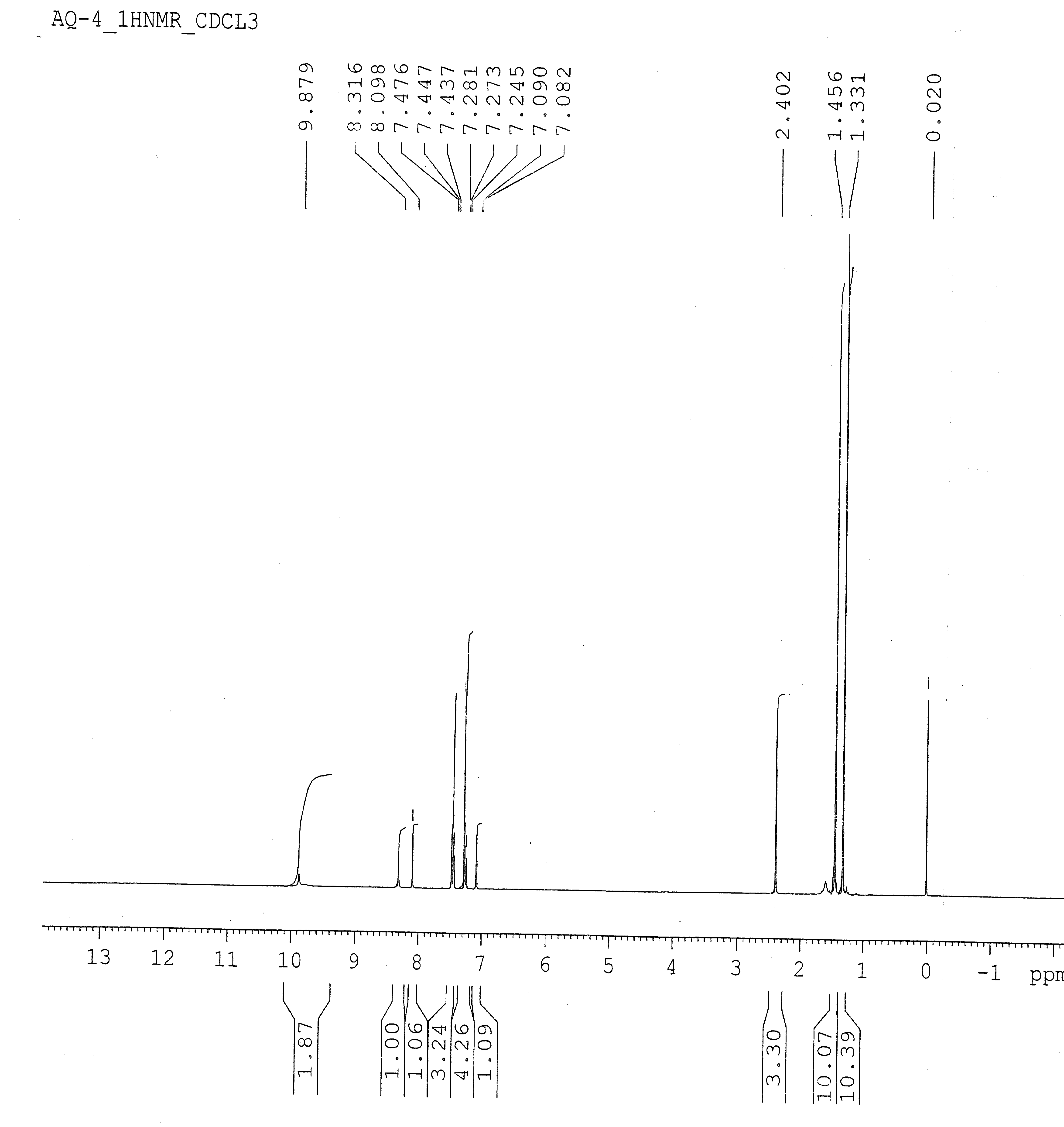


**Supplementary Figure S10:** ^1^H NMR spectra of compound **3d**

**Supplementary Figure S11:** ^13^C NMR spectra of compound **3d**

 **(3e)**

**Supplementary Figure S12:** ^1^H NMR spectra of compound **3e**

**Supplementary Figure S13:** ^13^C NMR spectra of compound **3e**

 **(3f)**

**Supplementary Figure S14:** ^1^H NMR spectra of compound **3f**

**Supplementary Figure S15:** ^13^C NMR spectra of compound **3f**

 **(3g)**


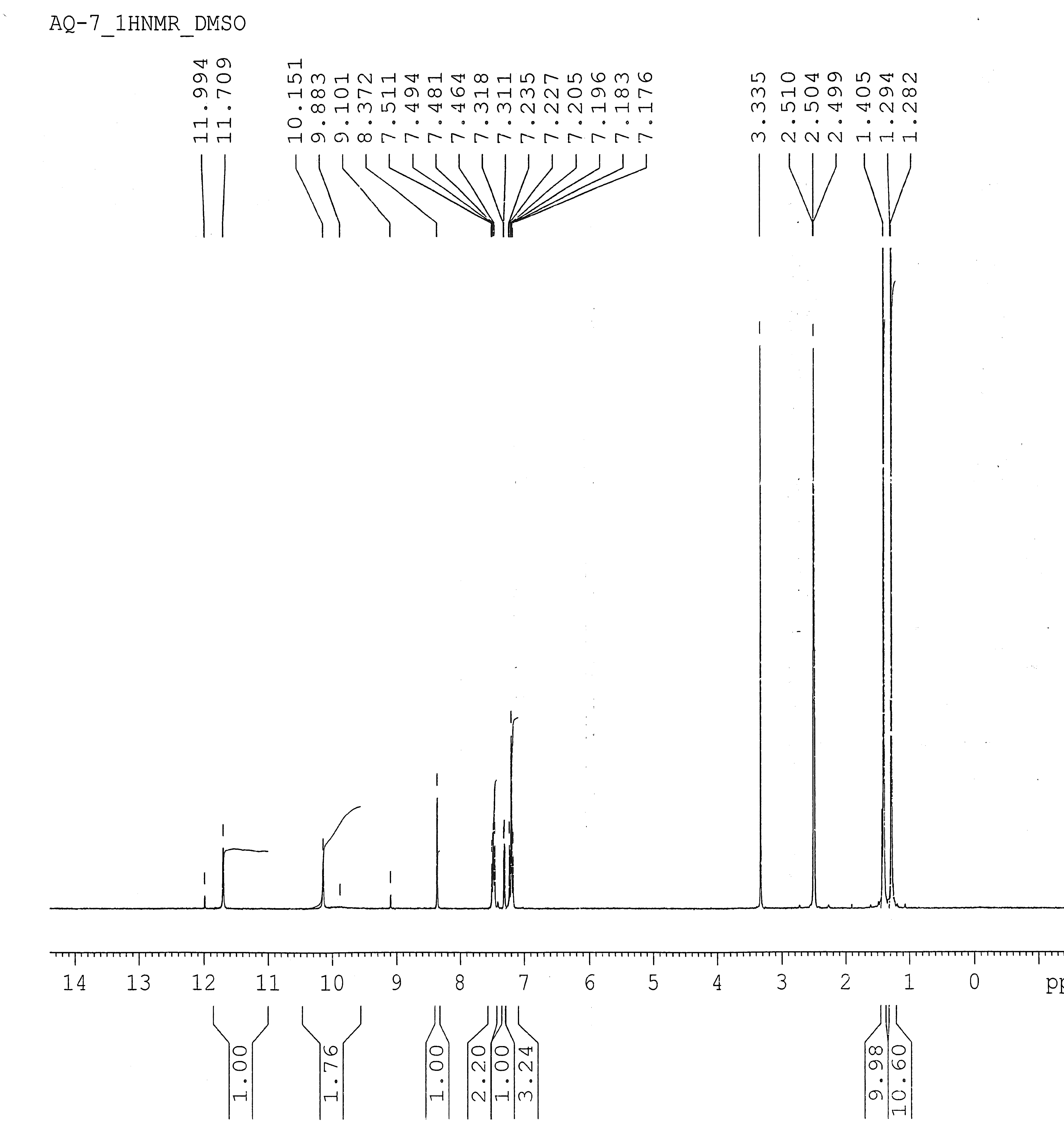


**Supplementary Figure S16:** ^1^H NMR spectra of compound **3g**

**Supplementary Figure S17:** ^13^C NMR spectra of compound **3g**

 **(3h)**


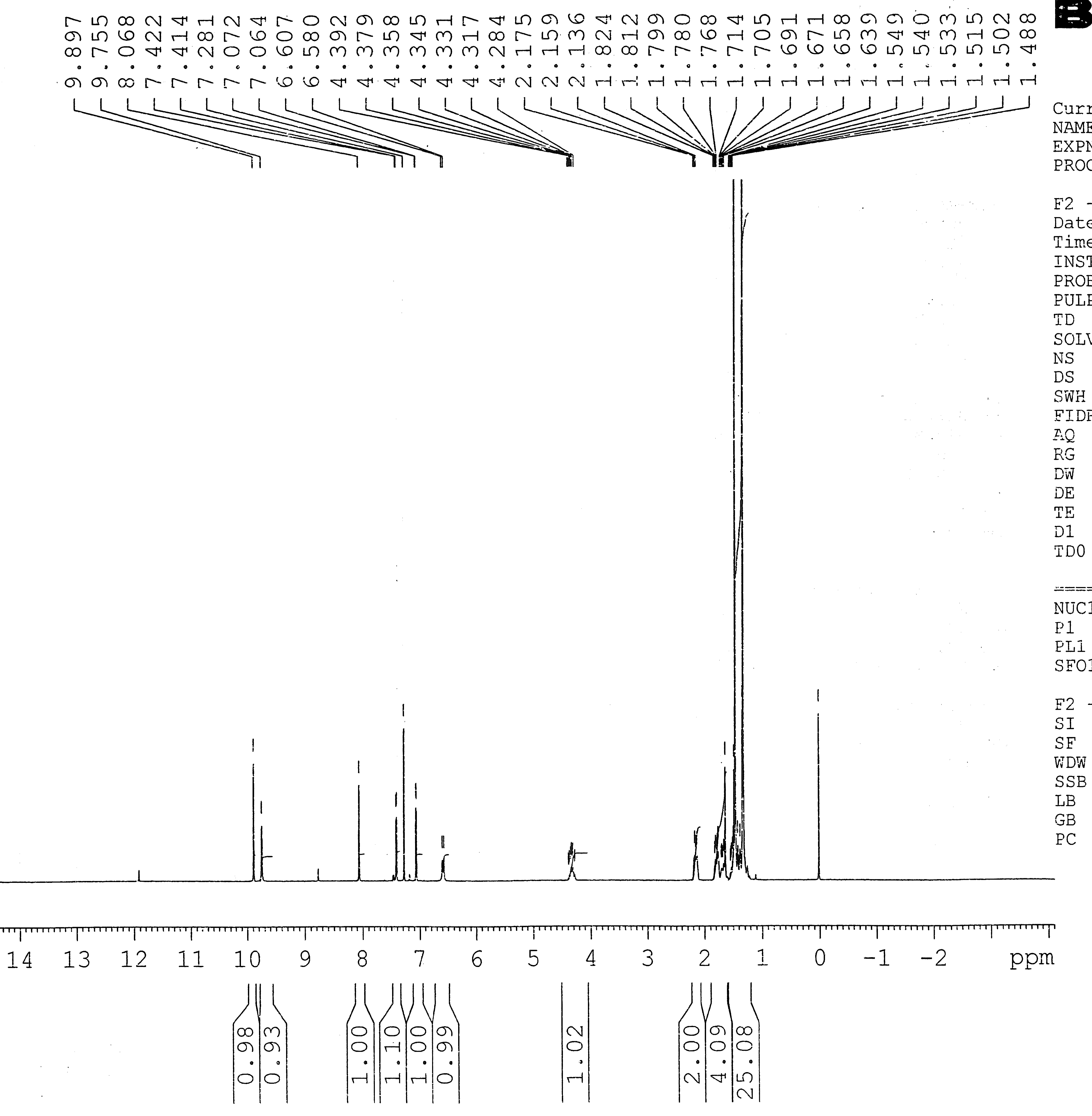


**Supplementary Figure S18:** ^1^H NMR spectra of compound **3h**

**Supplementary Figure S19:** ^13^C NMR spectra of compound **3h**

 **(3i)**

**Supplementary Figure S20:** ^1^H NMR spectra of compound **3i**

**Supplementary Figure S21:** ^13^C NMR spectra of compound **3i**

 **(3j)**

**Supplementary Figure S22:** ^1^H NMR spectra of compound **3j**

**Supplementary Figure S23:** ^13^C NMR spectra of compound **3j**

 **(3k)**

**Supplementary Figure S24:** ^1^H NMR spectra of compound **3k**

**Supplementary Figure S25:** ^13^C NMR spectra of compound **3k**

 **(3l)**

**Supplementary Figure S26:** ^1^H NMR spectra of compound **3l**

**
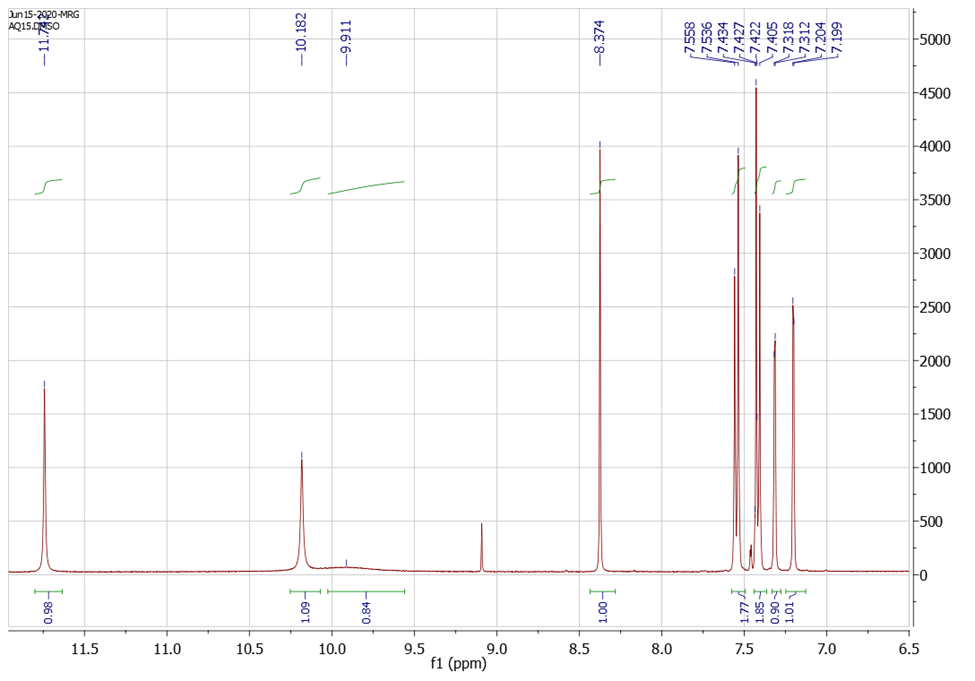
**

**Supplementary Figure S27: ^1^H NMR spectra of compound 3l (Aromatic region)**

**Supplementary Figure S28:** ^13^C NMR spectra of compound **3l**

 **(3m)**

**Supplementary Figure S29:** ^1^H NMR spectra of compound **3m**

**
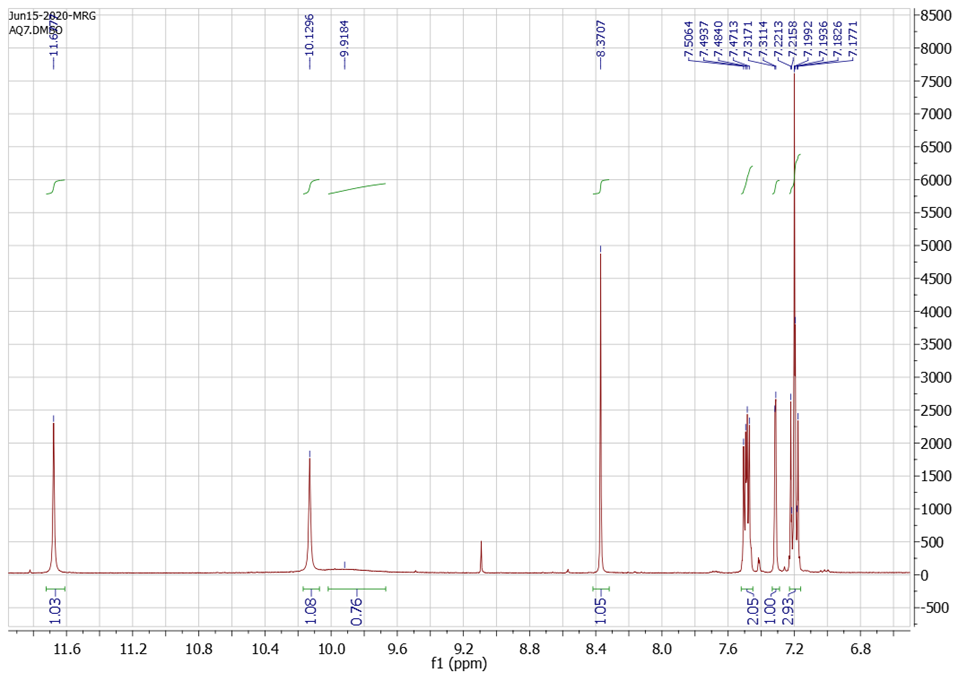
**

**Supplementary Figure S30: ^1^H NMR spectra of compound 3m**

**Supplementary Figure S31:** ^13^C NMR spectra of compound **3m**

 **(3n)**


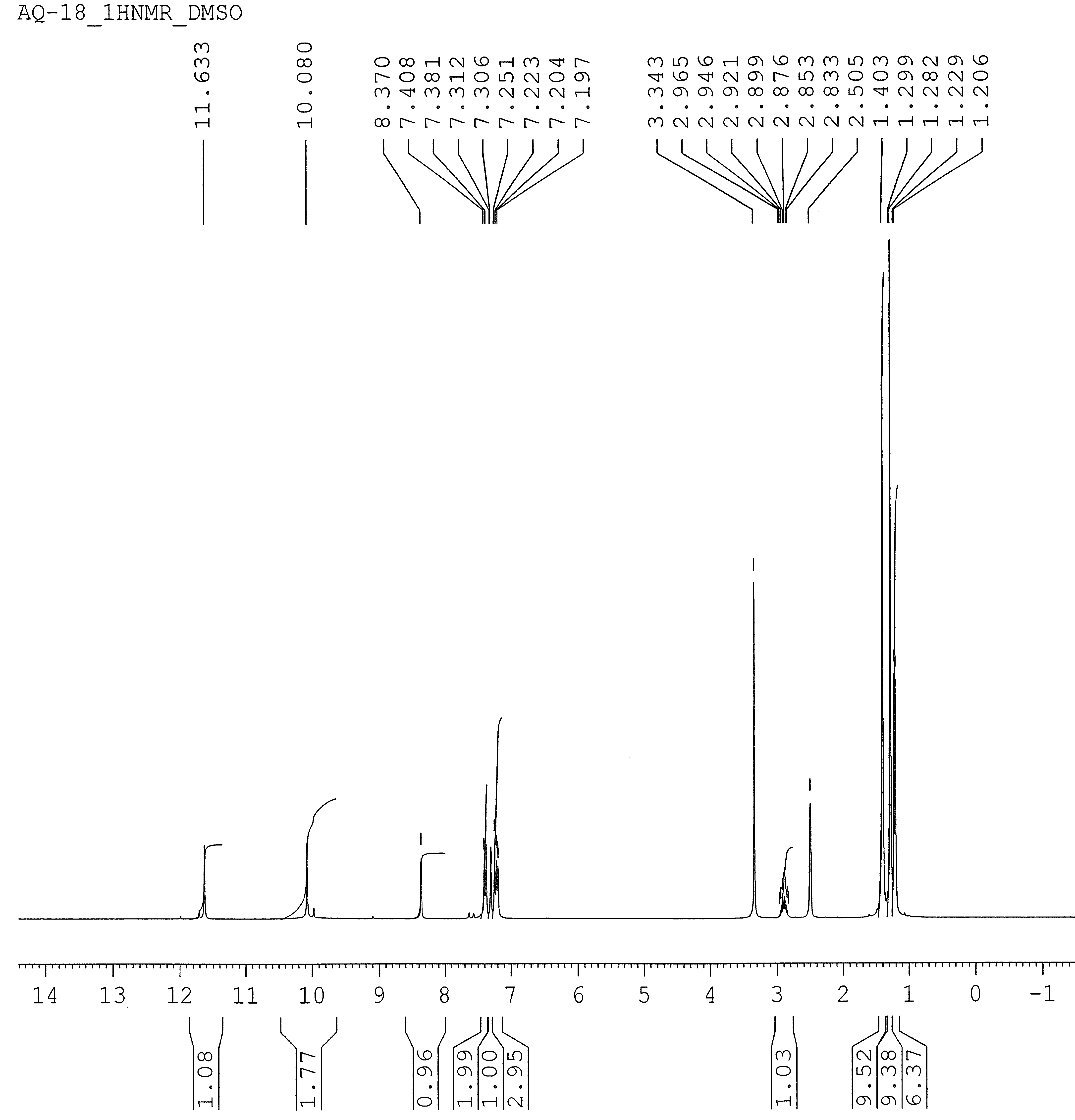


**Supplementary Figure S32:** ^1^H NMR spectra of compound **3n**

**Supplementary Figure S33:** ^13^C NMR spectra of compound **3n**

 **(3o)**

**Supplementary Figure S34:** ^1^H NMR spectra of compound **3o**

**Supplementary Figure S35:** ^13^C NMR spectra of compound **3o**
